# Enantioselective Synthesis of a Tricyclic, sp^3^‐Rich Diazatetradecanedione: an Amino Acid‐Based Natural Product‐Like Scaffold

**DOI:** 10.1002/chem.201905144

**Published:** 2020-02-19

**Authors:** Matthias Bischoff, Peter Mayer, Christian Meyners, Felix Hausch

**Affiliations:** ^1^ Compound Management and Screening Center (COMAS) Max Planck Institute of Molecular Physiology Otto-Hahn-Strasse 11 44227 Dortmund Germany; ^2^ Department of Chemistry Ludwig-Maximilians-University München Butenandtstrasse 5–13 81377 München Germany; ^3^ Department of Chemistry Institute of Chemistry and Biochemistry Darmstadt University of Technology Alarich-Weiss-Strasse 4 64287 Darmstadt Germany

**Keywords:** amino acids, diastereoselective epoxidation, FK506-binding protein, natural products, sp^3^−sp^2^ Negishi coupling

## Abstract

6‐, 7‐, and 8‐membered rings are assembled from a linear precursor by successive cyclisation reactions to construct a tricyclic diazatricyclo[6.5.1.0^4, 9^]‐tetradecanedione scaffold. Advanced building blocks based on d‐aspartic acid and l‐pyroglutamic acid were combined by a sp^3^−sp^2^ Negishi coupling. A carbamate‐guided *syn*‐diastereoselective epoxidation followed by an intramolecular epoxide opening allowed the construction of the piperidine ring. An efficient one‐pot hydroxyl‐group protection twofold deprotection reaction prepared the ground for the cyclisation to the bicycle. A final deprotection of the orthogonal protecting groups and lactamisation led to the novel, sp^3^‐rich tricycle. The final compound is a substrate mimic of peptidyl‐prolyl *cis‐trans* isomerases featuring a locked *trans*‐amide bond. Cheminformatic analysis of 179 virtual derivatives indicates favourable physicochemical properties and drug‐like characteristics. As proof of concept we, show a low micromolar activity in a fluorescence polarisation assay towards the FK506‐binding protein 12.

## Graphical abstract

**sp^3^‐Enriched scaffolds**: Using simple amino acids, an elaborated synthesis was designed to construct a rigid tricyclic scaffold consisting of six‐, seven‐ and eight‐membered rings.

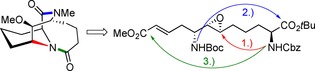

A recent scaffold analysis of more than 24 million organic compounds from the CAS Registry concluded that a ‘very small percentage of frameworks are found in a large percentage of compounds’. Concretely, more than 75 % of the compounds can be described by only 5 % of the most common frameworks. Thus, the structural diversity of the organic chemical space has been explored to a very limited extend.^1^

Obviously, in the context of medicinal chemistry, the question arises which kind of compounds have to be synthesized to find small molecule modulators of biological targets.^2^ In particular sp^3^‐rich, natural product‐like scaffolds have shifted into focus as new biologically active compounds with novel properties. Small molecule secondary metabolites have been selected and optimized during evolution to interact with biological targets and represent prevalidated chemical structures explored by nature.^3^ In accordance with that is the fact that natural products and natural product scaffolds continue to contribute to a large extent in the area of drug discovery and development. More than 50 percent of all new approved drugs in the time period 1981 to 2014 are natural product related.^4^

Representative examples for heterocyclic, sp^3^‐rich scaffolds are shown in Scheme 1. Furopyrane **A** was synthesized from a d‐ribose derivative by an intramolecular hetero Diels–Alder reaction.^5^ The key step for the construction of spiro lactam **B** consists of a domino nitrone formation/intramolecular [3+2] cycloaddition^6^ and phenol **C** was built by a domino Petasis/intramolecular hetero Diels–Alder reaction.^7^

**Scheme 1:**
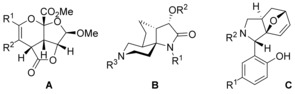
Examples of heterocyclic, sp^3^‐rich and natural product‐like scaffolds.

All three examples have in common that the complexity‐generating step consists of a multicomponent reaction, a cycloaddition or a combination of both. Undoubtedly, these are very elegant and efficient methods to establish molecular complexity. Nevertheless, there is a considerable relevant chemical space, which cannot be addressed by that means. The use of naturally occurring building blocks like amino acids benefit from the intrinsic chirality available in both enantiomeric forms. It offers the possibility to build up complex scaffolds with a maximum degree of flexibility regarding size, shape and stereochemistry. Here, we like to present a synthesis of a novel, sp^3^‐rich natural product‐like scaffold based on amino acids.

In the light of our general interest in sp^3^‐enriched scaffolds and in constrained amino acid analogues as the key to efficiently target protein–protein interactions, we have previously demonstrated that the conformationally preorganized diazabicyclo[4.3.1]decanone scaffold shows superior binding affinities and ligand efficiencies compared to more flexible motifs.^8^ In our quest to further rigidify this bioactive scaffold, we here describe a novel, highly rigidified diazatricyclo[6.5.1.0^4, 9^]tetradecanedione core, which can be synthesized in a stereochemically defined and substrate‐controlled manner using simple amino acids as chiral starting material.

Retrosynthetic analysis of **1**, based on an intramolecular epoxide opening and two amide bond formations, leads to epoxide **2**, which in turn is accessible from advanced vinyl iodide building block **3** and δ‐iodinated amino acid derivative **4** by a Negishi coupling (Scheme 2). **3** can be derived from d‐aspartic acid (**5**), whereas **4** can be derived from l‐pyroglutamic acid (**6**). The synthesis of **3** was accomplished in 7 steps (Scheme 3). Treatment of d‐aspartic acid (**5**) with thionyl chloride in methanol followed by *tert*‐butyloxycarbonyl (Boc) protection afforded methyl ester **8**.^9^ Borane reduction of the carboxylic acid yielded alcohol **9** in 68 % yield. Parikh–Doehring oxidation^10^ delivered an α‐amino aldehyde, which was directly subjected to Takai olefination conditions to give vinyl iodide **10** as a single *E*‐isomer in 46 % over 2 steps. Reduction of the methyl ester with DIBAL (diisobutylaluminium hydride) in toluene at −78 °C afforded the desired aldehyde **12** in 74 % yield along with the corresponding alcohol **11**, which could be reoxidized to the aldehyde in 83 % yield using IBX (2‐iodoxybenzoic acid) in DMSO. Finally, a Horner–Wadsworth–Emmons (HWE) reaction with methyl diethylphosphonoacetate delivered advanced vinyl iodide **3** in 79 % yield.

**Scheme 2:**
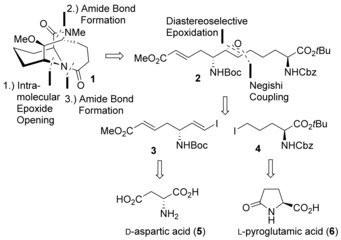
Retrosynthesis of the diazatricyclo[6.5.1.0^4, 9^]tetradecanedione motif **1**.

**Scheme 3:**
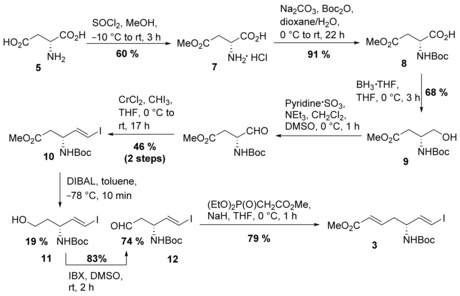
Synthesis of advanced vinyl iodide **3** starting from d‐aspartic acid.

For the completion of the synthesis of the tricyclic scaffold **1**, we coupled vinyl iodide **3** with **4** in a sp^3^−sp^2^ Negishi reaction in 71 % yield (Scheme 4).^11^ Compound **4** can be prepared in 4 steps from l‐pyroglutamic acid.8b Based on previous reports about stereoselective epoxidations of allylic carbamates in favour of the *syn*‐isomer,^12^ we were pleased to obtain a diastereomeric ratio of 4:1 and a yield of 79 % when treating alkene **13** with *m*CPBA (*meta*‐chloroperoxybenzoic acid). Hydrogenation of epoxide **2** with Pd/C as catalyst allowed the intramolecular opening of the epoxide and the formation of the piperidine ring, with simultaneous reduction of the double bond. Reprotection of the amine with benzyl chloroformate afforded **14** in 64 % yield over two steps. Formation of the second ring was accomplished by simultaneous triethylsilyl (TES) protection of the hydroxyl function and *t*Bu and Boc deprotection using TESOTf in lutidine,^13^ followed by lactamisation by means of HATU to give bicycle **15** in 74 % over two steps. The TES group was cleaved by TBAF (tetra‐*n*‐butylammonium fluoride) and the alcohol and the amide functionalities were methylated with MeI and silver(I) oxide as base in 74 % over two steps. Ultimately, the methyl ester in **16** was hydrolysed with lithium hydroxide, the carboxybenzyl (Cbz) group was cleaved with H_2_ and Pd/C, and the third ring was closed smoothly using HATU to provide the target tricycle **1** in 72 % yield over three steps.

**Scheme 4:**
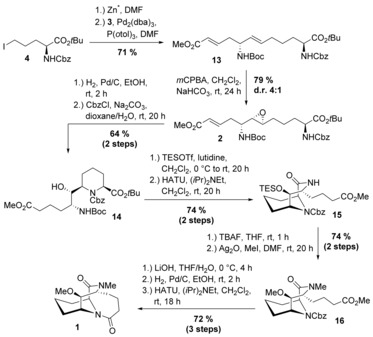
Synthesis of tricyclic scaffold **1**. HATU=hexafluorophosphate azabenzotriazole tetramethyl uronium.

Pipecolate derivatives such as **1** have been suggested to mimic substrates of peptidyl‐prolyl *cis*‐*trans* isomerases. To understand the precise architecture of this novel framework, we prepared crystals of **1**, which were suitable for single‐crystal X‐ray diffraction (Scheme 5 a). This showed an undistorted chair conformation of the piperidine ring featuring a *trans* conformation of the N1‐C1‐O1 amide bond. Importantly, the geometry of compound **1** perfectly matched the active conformation of the natural product FK506, when bound to FKBPs (Scheme 5 b).

**Scheme 5:**
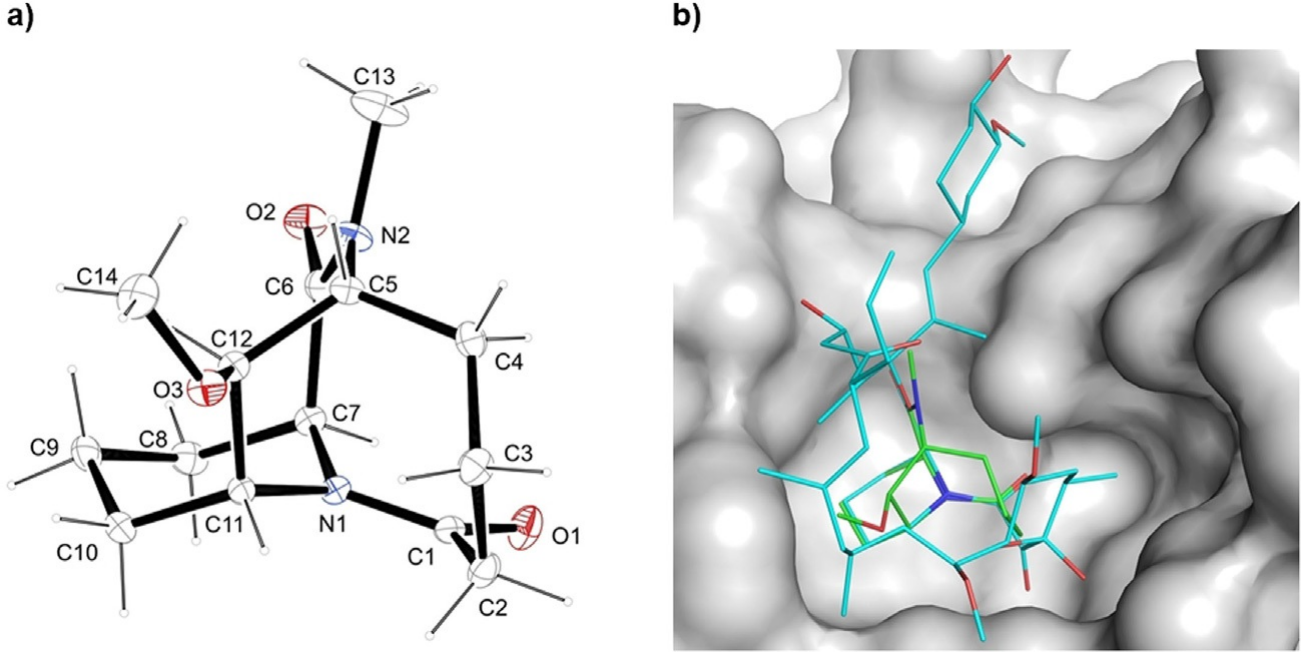
a) Crystal structure of tricyclic scaffold **1**. One molecule of H_2_O is omitted for clarity. b) Crystal structures of compound **1** (green) superimposed on FK506 (cyan) bound to the FK1 domain of FKBP51 (grey surface, PDB‐ID: 3O5R^15^).

This highly rigid ring system can be regarded as a structural scaffold with well‐defined vectors of the functional groups, that is, amide and methyl ether. Instead of the methyl groups (C13 and C14), these functionalities offer two independent attachment points for straightforward further derivatization. Additionally, this framework possesses a high degree of complexity, which is expressed through the fractional sp^3^ character (Fsp^3^=0.86) and the number of stereogenic centres (4), and should therefore be suitable for the exploration of drug‐relevant chemical space.^14^

To assess the molecular properties of this new scaffold we used the open‐access cheminformatic tool LLAMA (lead‐likeness and molecular analysis).^16^ Based on medicinal chemistry capping groups the tricycle was virtually decorated once or twice by amide alkylation/arylation, alcohol alkylation/arylation, esterification and carbamate formation resulting in 179 derivatives (Figure 1). The mean PMI (principal moment of inertia) coordinates (I1=0.416, I2=0.842), as indicated by the ‘+‘, show a substantial shift from the linear—disc‐like axis towards three‐dimensionality. 22 of the 179 derivatives (12 %) fall within the lead‐like space, whereas 152 of the 179 derivatives (85 %) fall within the Lipinski ‘rule of 5‘ space. Taken this data together, these derivatives have favourable physicochemical properties and occupy a drug‐like chemical space.

**Figure 1:**
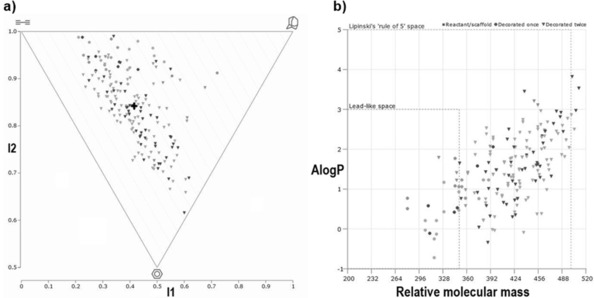
a) PMI plot and b) AlogP vs. molecular mass plot of 179 virtual derivatives of scaffold **1** based on the decoration of the hydroxyl group and/or the amide group.

We were interested to see if tricycle 1 can modulate emerging biological targets such as protein–protein interactions. As a first proof of concept, we tested binding of **1** to the FK506‐binding proteins FKBP12, FKBP51 and FKBP52,^17^ which have raised substantial interest as potential targets for neurological and psychiatric diseases (Figure 2).^18^ Indeed, **1** bound to FKBP12 with a *K*_i_ of 5±1 μm, making it one of the smallest FKBP12 ligands known.^19^ **1** did not bind to the larger FKBP51 or FKBP52 up to the solubility limits.

**Figure 2:**
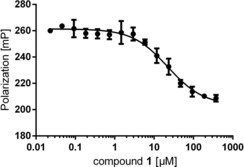
Binding of compound **1** to human FKBP12 (1 nm), determined by a fluorescence polarization^16^ using 0.5 nm of the TAMRA‐labelled compound [4.3.1]‐16 g8d as a tracer. The data points represent the mean of three independent replicates and the standard deviation is indicated by the error bars. The continuous line represents a least square fit to a competitive binding model yielding a *K*_i_ value of 5±1 μm for compound **1**.

In summary, we have developed an enantioselective synthesis of a tricyclic, sp^3^‐rich scaffold based on simple amino acids as starting material. d‐aspartic acid was elaborated in 7 steps into an advanced vinyl iodide building block. Sp^3^−sp^2^ Negishi coupling with a l‐pyroglutame derived building block and a diastereoselective epoxidation of the resulting alkene constituted the key steps of the synthesis. A cheminformatic analysis of 179 virtual derivatives of the novel tricycle, which were generated by the LLAMA tool, indicated favourable physicochemical properties and drug‐like characteristics. Finally, we showed the biological relevance of this novel scaffold by confirming an undistorted, locked *trans*‐conformation of the key amide bond and a low micromolar inhibition of the FK506‐binding protein 12.

## Experimental Section

### X‐ray crystallographic data

CCDC https://summary.ccdc.cam.ac.uk/structure-summary?doi=10.1002/chem.201905144 for compound **1** contains the supplementary crystallographic data for this paper. These data are provided free of charge by http://www.ccdc.cam.ac.uk/.

## Conflict of interest

The authors declare no conflict of interest.

## Supporting information

As a service to our authors and readers, this journal provides supporting information supplied by the authors. Such materials are peer reviewed and may be re‐organized for online delivery, but are not copy‐edited or typeset. Technical support issues arising from supporting information (other than missing files) should be addressed to the authors.

Supplementary
