# Supplementary material for: Enantioselective Synthesis of a Tricyclic, sp3‐Rich Diazatetradecanedione: an Amino Acid‐Based Natural Product‐Like Scaffold
Source: Chemistry. 2020 Feb 19;26(21):4677–81. doi: 10.1002/chem.201905144 (PMC7187416; doi:10.1002/chem.201905144)
Supplement: Supplementary file 1 — Supplementary [file CHEM-26-4677-s001.pdf]

Chemistry–A European Journal

Supporting Information

Enantioselective Synthesis of a Tricyclic, sp^3-Rich Diazatetradecanedione: an Amino Acid-Based Natural Product-Like Scaffold

Matthias Bischoff,[a] Peter Mayer,[b] Christian Meyners,[c] and Felix Hausch*[c]

Enantioselective Synthesis of a tricyclic, sp^3-rich Diazatetradecanedione: an Amino Acid-based Natural Product-like Scaffold

Matthias Bischoff[a], Peter Mayer[b], Christian Meyners[c] and Felix Hausch[c*]

[a] Compound Management and Screening Center (COMAS), Max Planck Institute of Molecular Physiology, Otto-Hahn-Strasse 11, 44227 Dortmund, Germany

[b] Department of Chemistry, Ludwig-Maximilians-University München, Butenandtstrasse 5-13, 81377 München, Germany

[c] Department of Chemistry, Institute of Chemistry and Biochemistry, Darmstadt University of Technology, Alarich-Weiss-Strasse 4, 64287 Darmstadt, Germany

Supporting Information

Table of Contents

Biochemical FKBP12 Binding Assay S2

General Experimental S3

Syntheses and Analytical Data of the Compounds S4

^{1}H- and ^{13}C-Spectra of the Compounds S17

Crystallographic Data S29

Biochemical FKBP12 Binding Assay

The Ki-value of compound 1 was determined by using a fluorescence polarization assay as described earlier. Therefore, a serial dilution of compound 1 or FK506 in assay buffer (20 mM HEPES pH 8.0, 20 mM NaCl, 0.002% Triton X-100) was placed in a 384 well micro titer plate and supplemented with a mixture of purified FKBP12 and the fluorescent tracer [4.3.1]-16g yielding final concentrations of 1 nM and 0.5 nM. After an incubation of 30 minutes at 25 °C the fluorescence polarization was determined on a mirco plate reader using an excitation wavelength of 535 nm and an emission wavelength of 590 nm. The obtained data was plotted against the compound concentration and fitted to a competitive binding model yielding the Ki-value of the compounds.

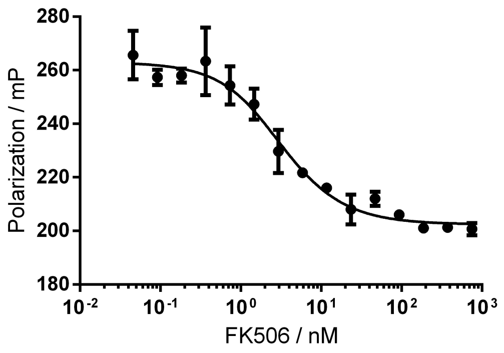

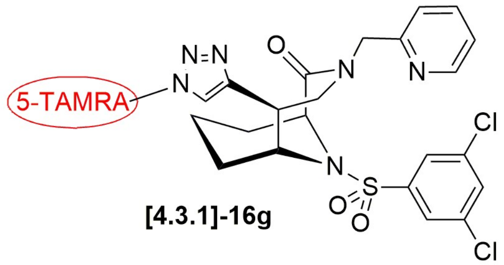

[4.3.1]-16g

General Experimental

Reactions were performed in heatgun-dried flasks under an argon atmosphere. All reagents purchased from commercial sources were used directly without further purification.

^{1}H and ^{13}C-NMR spectra were recorded at the Department of Chemistry and Pharmacy, Ludwig Maximilians University München on a Bruker AC 300, a Bruker XL 400, or a Bruker AMX 600 at room temperature unless otherwise specified. Chemical shifts are given in ppm (δ). Residual peaks of the deuterated solvents indicated were used as internal standard. The coupling constants (*J*) are given in Hertz (Hz). The following abbreviations are used for the characterization of the multiplicity of the signals: singlet (s), singlet broad (s_{br}), doublet (d), triplet (t), quartet (q) multiplet (m) and centered multiplet (m_c).

Mass spectra (*m/z*) were obtained on a Thermo Finnigan LCQ DECA XP Plus mass spectrometer at the Max Planck Institute of Psychiatry München, while the high resolution mass spectrometry was carried out at Max Planck Institute of Biochemistry München on a Bruker micrOTOF LC mass spectrometer.

Thin-layer chromatography (TLC) was performed on precoated silica gel F-254 plates from Merck. The spots were visualized by UV light and/or by staining of the TLC plate with potassium permanganate stain (1.5 g $KMnO_4$, 10 g K_2CO_3, 1.25 mL 10 % NaOH in 200 mL H_2O) followed, if necessary, by heating with a heat gun.
For column chromatography, silica gel 60 from Merck with a particle size of 0.040-0.063 mm was used.

Syntheses and Analytical Data of the Compounds

D-Aspartic acid 4-methyl ester hydrochloride (7)

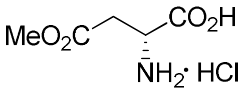

Methanol (210 mL) was cooled to −20 °C and $SOCl_2$ (31.0 mL, 425 mmol, 1.4 equiv.) was added dropwise over 45 min. D-Aspartic acid (40.0 g, 300 mmol) was added over 5 min, the cooling bath was removed and stirred for 3 h at room temperature. Et_2O (600 mL) was added and the mixture cooled to −20 °C. The resulting solid was filtered off, washed with Et_2O (200 mL) and dried under reduced pressure to give the title compound (33.0 g, 180 mmol, 60 %) as a colorless solid.

^{1}H-NMR (400 MHz, DMSO-d_6): δ = 2.98 (d, J = 5.6, 1 H, CH_A), 2.99 (d, J = 5.6, 1 H, CH_B), 3.64 (s, 3 H, CO_2Me), 4.16 (t, J = 5.6, 1 H, CH), 8.64 (s_{br}, 2 H, CO_2H, HCl).

^{13}C-NMR (100 MHz, DMSO-d_6): δ = 34.05, 48.47, 52.05, 169.6, 169.8.

HRMS (ESI) for $C_5H_9NO_4$: calcd. 148.0610 $[M+H]^+$, found 148.0624.

***N*-Boc-D-aspartic acid 4-methyl ester (8)**

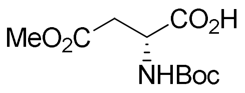

To a solution of Na_2CO_3 (19.1 g, 180 mmol, 1.0 equiv.) in dioxane/H_2O (540 mL, 2:1) at 0 °C was added **7** (33.0 g, 180 mmol, 1.0 equiv.). After the CO_2 evolution had ceased (15 min), Na_2CO_3 (19.1 g, 180 mmol, 1.0 equiv.) and Boc_2O (43.2 g, 198 mmol, 1.1 equiv.) were successively added. The mixture was stirred for 1 h at 0 °C and for 21 h at room temperature. The dioxane was removed under reduced pressure, the residue was poured in ice-water (350 mL) and washed with Et_2O (250 mL). The aqueous phase was acidified with sat. aq. $NaHSO_4$-solution (pH 2.5) and extracted with Et_2O (3 × 250 mL). The combined organic phases were washed with H_2O (250 mL), dried over $MgSO_4$, and the solvent was

removed under reduced pressure to give the title compound (91 %, 40.5 g, 164 mmol), as a colorless solid.

^{1}H-NMR (300 MHz, $CDCl_3$): δ = 1.44 (s, 9 H, 3 × CH_3), 2.84 (dd, *J* = 4.8, 17.1 Hz, 1 H, CH_A), 3.03 (dd, *J* = 3.6, 17.1 Hz, 1 H, CH_B), 3.71 (s, 3 H, CO_2Me), 4.61 (m, 1 H, CH), 5.56 (d_{br}, *J* = 8.4 Hz, 1 H, NH) 7.20 (s_{br}, 1 H, CO_2H).

^{13}C-NMR (75.5 MHz, $CDCl_3$): δ = 28.41, 36.55, 49.91, 52.28, 80.66, 155.8, 171.7, 175.4.

HRMS (ESI) for $C_{10}H_{17}NO_6$: calcd. 270.0954 $[M+Na]^+$, found 270.0976.

Methyl (3*R*)-3-{[(*tert*-butoxy)carbonyl]amino}-4-hydroxybutanoate (9)

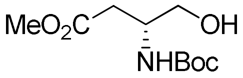

A solution of **8** (16.5 g, 66.7 mmol) in THF (67 mL) was added dropwise over 1 h to a solution of BH_3·THF (1 M in THF, 200 mL, 200 mmol, 3.0 equiv.) at 0 °C. It was stirred for further 2 h at 0 °C and sat. aq. NH_4Cl solution (250 mL) was carefully added in portions over 1 h. The mixture was extracted with EtOAc (2 × 250 mL), the combined organic phases were washed with sat. aq. NaCl solution (250 mL), dried over $MgSO_4$, and the solvent was removed under reduced pressure. Column chromatography on SiO_2 (cyclohexane/EtOAc 1:1 → 1:2) afforded the title compound (10.6 g, 45.4 mmol, 68 %) as a colorless oil.

R_f: 0.38 (Cyclohexane/EtOAc 1:1, $KMnO_4$)

^{1}H-NMR (400 MHz, DMSO-d_6): δ = 1.39 (s, 9 H, 3 × CH_3), 2.30 (dd, *J* = 8.4, 15.2 Hz, 1 H, CH_A), 2.52 (dd, *J* = 5.2, 15.2 Hz, 1 H, CH_B), 3.18−3.25 (m, 1 H, CH_A), 3.32−3.41 (m, 1 H, CH_B), 3.56 (s, 3 H, CO_2Me), 3.71−3.83 (m, 1 H, NH), 4.74 (t, *J* = 5.6 Hz, 1 H, CH), 6.61 (d, *J* = 8.8 Hz, 1 H, OH).

^{13}C-NMR (100 MHz, DMSO-d_6): δ = 28.18, 36.12, 49.60, 51.25, 62.84, 77.64, 155.0, 171.7.

MS (ESI): *m/z* (%) = 134.0 (45) [M-Boc+H]$^+$, 233.8 (21) [M+H]$^+$, 256.1 (14) [M+Na]$^+$, 366.7 (100) [2M-Boc+H]$^+$, 488.8 (16) [2M+Na]$^+$.

HRMS (ESI) for $C_{10}H_{19}NO_5$: calcd. 256.1161 [M+Na]$^+$, found 256.1174.

Methyl (3*R*,4*E*)-3-{[(*tert*-butoxy)carbonyl]amino}-5-iodopent-4-enoate (10)

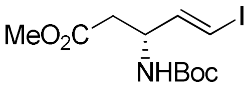

A solution of **9** (7.57 g, 32.5 mmol) and NEt_3 (27.0 mL, 195 mmol, 6.0 equiv.) in CH_2Cl_2 (65 mL) was cooled to 0 °C and a suspension of pyridine·SO_3 (31.0 g, 195 mmol, 6.0 equiv.) in DMSO (65 mL) was added. After stirring for 1 h at 0 °C, ice water (250 mL) was added and it was extracted with CH_2Cl_2 (3 × 200 mL). The combined organic phases were successively washed with 10% citric acid solution (3 × 100 mL), H_2O (3 × 100 mL), sat. aq. $NaHCO_3$ solution (100 mL), sat. aq. NaCl solution (100 mL), dried over $MgSO_4$, and the solvent was removed under reduced pressure to afford the aldehyde (6.75 g, 29.2 mmol, 90%) as an orange oil, which was used without further purification in the next step.

A solution of the aldehyde (6.75 g, 29.2 mmol) and CHI_3 (11.5 g, 38.0 mmol, 1.3 equiv.) in THF (73 mL) was added dropwise over 0.5 h to a suspension of $CrCl_2$ (14.3 g, 117 mmol, 4.0 equiv.) in THF (146 mL) at 0 °C. The cooling bath was removed and it was stirred for further 17 h at room temperature. H_20 (250 mL) was added and it was extracted with Et_2O (3 × 150 mL). The combined organic phases were washed with 1 M $Na_2S_2O_3$ solution (150 mL), H_20 (150 mL), dried over $MgSO_4$, and the solvent was removed under reduced pressure. Column chromatography on SiO_2 (cyclohexane/EtOAc 8:1 → 4:1) afforded the title compound (5.26 g, 14.8 mmol, 46 % over 2 steps) as a colorless oil, which solidified upon standing at 4 °C.

R_f: 0.60 (Cyclohexane/EtOAc 4:1, $KMnO_4$)

^{1}H-NMR (400 MHz, $CDCl_3$): δ = 1.43 (s, 9 H, 3 × CH_3), 2.60 (dd, *J* = 2.0, 5.6 Hz, 2 H, CH_2), 3.70 (s, 3 H, CO_2Me), 4.47 (s_{br}, 1 H, CH), 5.26 (s_{br}, 1 H, NH), 6.36 (dd, *J* = 1.2, 14.4 Hz, 1 H, CH), 6.54 (dd, *J* = 6.0, 14.4 Hz, 1 H, CH).

^{13}C-NMR (100 MHz, $CDCl_3$): δ = 28.48, 38.58, 52.09, 77.36, 78.57, 80.16, 144.4, 155.0, 171.3.

MS (ESI): *m/z* (%) = 255.9 (100) $[M\text{-}Boc+H]^+$, 377.9 (18) $[M+Na]^+$, 610.5 (54) $[2M\text{-}Boc+H]^+$, 732.6 (10) $[2M+Na]^+$.

HRMS (ESI) for $C_{11}H_{18}INO_4$: calcd. 378.0178 $[M+Na]^+$, found 378.0175.

***tert*-Butyl N-[(1*E*,3*R*)-5-hydroxy-1-iodopent-1-en-3-yl]carbamate (11)**
***tert*-Butyl N-[(1*E*,3*R*)-1-iodo-5-oxopent-1-en-3-yl]carbamate (12)**

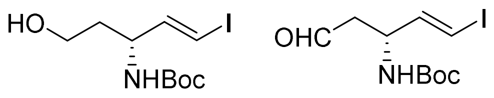

To a solution of **10** (5.26 g, 14.8 mmol) in toluene (74 mL) at −78 °C was added dropwise over 20 min a solution of DIBAL (1 M in toluene, 29.6 mL, 29.6 mmol, 2.0 equiv.). After stirring for further 10 min at −78 °C, $Na_2SO_4 \cdot 10\ H_20$ (20 g) was added, the cooling bath was removed and it was allowed to reach room temperature. The reaction mixture was filtered over Celite, washed with EtOAc and the solvent was removed under reduced pressure. Column chromatography on SiO_2 (cyclohexane/EtOAc 4:1 → 1:1) afforded the aldehyde (3.58 g, 11.0 mmol, 74 %) as slightly yellow oil, and the alcohol (905 mg, 2.77 mmol, 19 %) as slightly yellow oil.

Alcohol **11**:

R_f: 0.37 (Cyclohexane/EtOAc 2:1, $KMnO_4$)

MS (ESI): *m/z* (%) = 227.9 (100) $[M\text{-}Boc+H]^+$, 349.9 (18) $[M+Na]^+$, 554.5 (68) $[2M\text{-}Boc+H]^+$, 676.6 (10) $[2M+Na]^+$.

HRMS (ESI) for $C_{10}H_{18}INO_3$: calcd. 350.0229 $[M+Na]^+$, found 350.0238.

Aldehyde **12**:

R_f: 0.54 (Cyclohexane/EtOAc 2:1, $KMnO_4$)

^{1}H-NMR (300 MHz, $CDCl_3$): δ = 1.43 (s, 9 H, 3 × CH_3), 2.74 (dd, *J* = 1.2, 6.0 Hz, 2 H, CH_2), 4.48−4.62 (m, 1 H, CH), 4.95 (s_{br}, 1 H, NH), 6.38 (dd, *J* = 1.2, 14.7 Hz, 1 H, CH), 6.56 (dd, *J* = 6.6, 14.7 Hz, 1 H, CH), 9.71−9.75 (m, 1 H, CHO).

^{13}C-NMR (75.5 MHz, $CDCl_3$): δ = 27.07, 28.46, 47.89, 78.75, 80.40, 144.3, 154.9, 199.6.

MS (ESI): *m/z* (%) = 225.9 (26) $[M\text{-}Boc+H]^+$, 269.8 (100) $[M\text{-}t\text{Bu}+H]^+$, 325.7 (8) $[M+H]^+$.

HRMS (ESI) for $C_{10}H_{16}INO_3$: calcd. 380.0335 $[M+MeOH+Na]^+$, found 380.0346.

***tert*-Butyl N-[(1*E*,3*R*)-1-iodo-5-oxopent-1-en-3-yl]carbamate (12)**

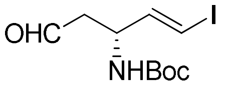

To a solution of **11** (2.92 g, 8.93 mmol) in DMSO (45 mL) was added IBX (3.75 g, 13.4 mmol, 1.5 equiv.) and the suspension was stirred for 16 h at room temperature. H_2O (100 mL) was added, it was filtered over Celite and washed with Et_2O. The filtrate was extracted with Et_2O (3 × 100 mL), the combined organic phases were washed with sat. aq. NaCl solution (100 mL), dried over $MgSO_4$, and the solvent was removed under reduced pressure. Column chromatography on SiO_2 (cyclohexane/EtOAc 3:1) afforded the title compound (2.40 g, 7.38 mmol, 83 %) as a slightly yellow oil.

R_f: 0.54 (Cyclohexane/EtOAc 2:1, $KMnO_4$)

^{1}H-NMR (300 MHz, $CDCl_3$): δ = 1.43 (s, 9 H, 3 × CH_3), 2.74 (dd, *J* = 1.2, 6.0 Hz, 2 H, CH_2), 4.48−4.62 (m, 1 H, CH), 4.95 (s_{br}, 1 H, NH), 6.38 (dd, *J* = 1.2, 14.7 Hz, 1 H, CH), 6.56 (dd, *J* = 6.6, 14.7 Hz, 1 H, CH), 9.71−9.75 (m, 1 H, CHO).

^{13}C-NMR (75.5 MHz, $CDCl_3$): δ = 27.07, 28.46, 47.89, 78.75, 80.40, 144.3, 154.9, 199.6.

MS (ESI): *m/z* (%) = 225.9 (26) $[M\text{-}Boc+H]^+$, 269.8 (100) $[M\text{-}t\text{Bu}+H]^+$, 325.7 (8) $[M+H]^+$.

HRMS (ESI) for $C_{10}H_{16}INO_3$: calcd. 380.0335 [M+MeOH+Na]$^+$, found 380.0346.

Methyl (2*E*,5*R*,6*E*)-5-(((*tert*-butoxy)carbonyl)amino)-7-iodohepta-2,6-dienoate (3)

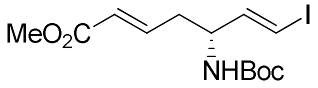

To a solution of Methyl diethylphosphonoacetate (674 mL, 3.70 mmol, 1.2 equiv.) in THF (15 mL) at 0 °C was added NaH (148 mg, 60 %, 3.70 mmol, 1.2 equiv.) and it was stirred for 0.5 h at 0 °C. Subsequently a solution of **12** (1.00 g, 3.08 mmol) in THF (2 mL) was added dropwise, and stirring was continued for 1 h at 0 °C. Sat. aq. NH_4Cl solution (50 mL) was added, the aqueous phase was extracted with EtOAc (2 × 50 mL), the combined organic phases were dried over $MgSO_4$, and the solvent was removed under reduced pressure. Column chromatography on SiO_2 (cyclohexane/EtOAc 4:1) afforded the title compound (926 mg, 2.43 mmol, 79 %) as a colorless oil.

R_f: 0.52 (Cyclohexane/EtOAc 3:1, $KMnO_4$)

^{1}H-NMR (600 MHz, $CDCl_3$): δ = 1.43 (s, 9 H, 3 × CH_3), 2.37−2.51 (m, 2 H, CH_2), 3.73 (s, 3 H, CO_2Me), 4.29 (s_{br}, 1 H, CH), 4.54 (s_{br}, 1 H, NH), 5.90 (dt, *J* = 1.8, 15.6 Hz, 1 H, CH), 6.34 (dd, *J* = 1.2, 14.4 Hz, 1 H, CH), 6.47 (dd, *J* = 6.0, 14.4 Hz, 1 H, CH), 6.85 (dt, *J* = 7.2, 15.6 Hz, 1 H, CH).

^{13}C-NMR (125 MHz, $CDCl_3$): δ = 28.44, 37.42, 51.75, 53.89, 78.44, 80.27, 124.6, 143.3, 144.9, 154.9, 166.4.

MS (ESI): *m/z* (%) = 281.8 (100) [M-Boc+H]$^+$, 403.9 (12) [M+Na]$^+$, 662.7 (36) [2M-Boc+H]$^+$.

HRMS (ESI) for $C_{13}H_{20}INO_4$: calcd. 404.0335 [M+Na]$^+$, found 404.0340.

12-*tert*-Butyl 1-methyl (2*E*,5*R*,6*E*,11*S*)-11-{[(benzyloxy)carbonyl]amino}-5-{[(*tert*-butoxy)carbonyl]amino}dodeca-2,6-dienedioate (13)

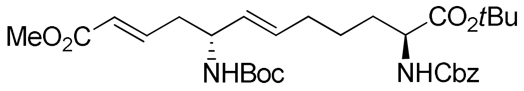

To a suspension of Zn powder (1.33 g, 20.3 mmol, 6.0 equiv.) in DMF (1.7 mL) was added 1,2-Dibromomethane (88 μL, 1.02 mmol, 0.3 equiv.) and it was stirred for 0.5 h at 60 °C. After cooling to room temperature, TMSCl (26 μL, 0.203 mmol, 0.06 equiv.) was added and stirring was continued for further 0.5 h at room temperature. Then *tert*-Butyl-(*S*)-2-(benzyloxycarbonylamino)-5-iodopentanoate (**4**) (1.47 g, 3.39 mmol, 1.0 equiv.) in DMF (1.7 mL) was added and the reaction mixture was stirred for 0.5 h at 35 °C. After cooling to room temperature, $Pd_2(dba)_3$ (62 mg, 0.068 mmol, 0.02 equiv), $P(otol)_3$ (83 mg, 0.271 mmol, 0.08 equiv.), and **3** (970 mg, 2.54 mmol, 0.75 equiv.) in DMF (0.5 mL) were added and it was stirred for 18 h at room temperature. The resulting green suspension was filtered over Celite and washed with EtOAc (100 mL). The filtrate was washed with sat. aq. NaCl solution (3 × 100 mL), dried over $MgSO_4$ and the solvent was evaporated under reduced pressure. Column chromatography on SiO_2 (cyclohexane/EtOAc 4:1) afforded the title compound (1.01 g, 1.80 mmol, 71 %) as yellow oil.

R_f: 0.33 (Cyclohexane/EtOAc 3:1, $KMnO_4$)

^{1}H-NMR (600 MHz, $CDCl_3$): δ = 1.33−1.41 (m, 2 H, CH_2), 1.43 (s, 9 H, 3 × CH_3), 1.45 (s, 9 H, 3 × CH_3), 1.57−1.65 (m, 1 H, CH_A), 1.72−1.81 (m, 1 H, CH_B), 1.99−2.11 (m, 2 H, CH_2), 2.36−2.47 (m, 2 H, CH_2), 3.71 (s, 3 H, CO_2Me), 4.18−4.28 (m 2 H, 2 × CH), 4.54 (s_{br}, 1 H, NH), 5.10 (s, 2 H, CH_2), 5.32 (d, *J* = 8.4 Hz, 1 H, NH), 5.36 (dd, *J* = 6.0, 15.6 Hz, 1 H, CH), 5.55 (dt, *J* = 6.0, 15.6 Hz, 1 H, CH), 5.86 (dt, *J* = 7.2, 15.6 Hz, 1 H, CH), 6.87 (dt, *J* = 6.0, 15.6 Hz, 1 H, CH), 7.29−7.37 (m, 5 H, 5 × Ar-H).

^{13}C-NMR (125 MHz, $CDCl_3$): δ = 24.70, 28.22, 28.55, 31.86, 32.51, 38.44, 51.34, 51.69, 54.33, 67.05, 79.74, 82.22, 123.8, 128.3, 128.3, 128.7, 130.1, 131.5, 136.6, 144.9, 155.2, 156.0, 166.8, 171.7.

MS (ESI): *m/z* (%) = 405.1 (24) $[M\text{-Boc-}t\text{Bu+H}]^+$, 461.1 (100) $[M\text{-Boc+H}]^+$, 527.1 (12) $[M\text{-}t\text{Bu+Na}]^+$, 583.1 (48) $[M+Na]^+$.

HRMS (ESI) for $C_{30}H_{44}N_2O_8$: calcd. 561.3176 $[M+Na]^+$, found 561.3220.

Methyl (2*E*,5*R*)-5-[(2*S*,3*S*)-3-[(4*S*)-4-{[(benzyloxy)carbonyl]amino}-5-(*tert*-butoxy)-5-oxopentyl]oxiran-2-yl]-5-{[(*tert*-butoxy)carbonyl]amino}pent-2-enoate (2)

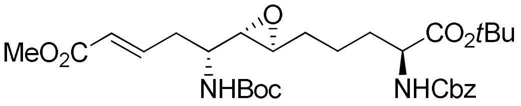

To a solution of **13** (367 mg, 0.654 mmol) in CH_2Cl_2 (6 mL) was added $NaHCO_3$ (82 mg, 0.981 mmol, 1.5 equiv.) and *m*CPBA (70 %, 177 mg, 0.719 mmol, 1.1 equiv.) and the suspension was stirred for 24 h at room temperature. CH_2Cl_2 (50 mL) was added and the organic phase was washed with sat. aq. $NaHCO_3$ solution (2 × 50 mL), dried over $MgSO_4$, and the solvent was evaporated under reduced pressure. Column chromatography on SiO_2 (cyclohexane/EtOAc 3:1 → 2:1) afforded the title compound (299 mg, 0.518 mmol, 79 %) as colorless oil.

The ratio of the two diastereomeric epoxides is 4:1.

R_f: 0.43 (Cyclohexane/EtOAc 2:1, $KMnO_4$)

^{1}H-NMR (600 MHz, $CDCl_3$): δ = 1.39−1.43 (m, 9 H, 3 × CH_3), 1.46 (s, 9 H, 3 × CH_3), 1.47−1.88 (m, 6 H, 3 × CH_2), 2.35−2.56 (m, 2 H, CH_2), 2.70−2.83 (m, 2 H, 2 × CH), 3.72 (s, 2.4 H, CO_2Me), 3.72 (s, 0.6 H, CO_2Me), 3.98−4.05 (m, 1 H, CH), 4.20−4.27 (m, 1 H, CH), 4.49 (d, *J* = 8.4 Hz, 1 H, NH), 5.08−5.12 (m, 2 H, CH_2), 5.31−5.37 (m, 1 H, NH), 5.91 (dt, *J* = 1.2, 15.6 Hz, 1 H, CH), 6.93 (dt, *J* = 7.2, 15.6 Hz, 1 H, CH), 7.29−7.38 (m, 5 H, 5 × Ar-H).

^{13}C-NMR (125 MHz, $CDCl_3$): δ = 21.83, 28.15, 28.39, 31.01, 32.62, 36.54, 48.33, 51.70, 54.37, 55.54, 58.23, 59.20, 67.04, 82.31, 124.2, 128.2, 128.3, 128.7, 136.5, 144.0, 155.5, 156.0, 166.0, 171.5.

MS (ESI): *m/z* (%) = 421.1 (28) $[M\text{-}Boc\text{-}tBu+H]^+$, 465.0 (100) $[M\text{-}2tBu+H]^+$, 520.8 (52) $[M\text{-} tBu+H]^+$, 599.1 (36) $[M+Na]^+$.

HRMS (ESI) for $C_{30}H_{44}N_2O_9$: calcd. 577.3125 $[M+H]^+$, found 577.3126.

1-Benzyl 2-*tert*-butyl (2*S*,6*R*)-6-[(1*S*,2*R*)-2-{[(*tert*-butoxy)carbonyl]amino}-1-hydroxy-6-methoxy-6-oxohexyl]piperidine-1,2-dicarboxylate (14)

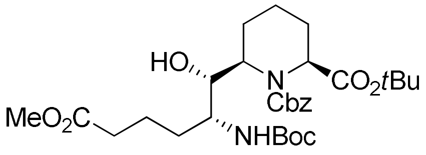

A suspension of **2** (282 mg, 0.489 mmol) and Pd/C (28 mg, 10 wt. %) in EtOH (5 mL) was stirred for 2 h at room temperature under a H_2-atmosphere. The reaction mixture was filtered over Celite, washed with EtOAc and the solvent was evaporated under reduced pressure to give a colorless, crystalline solid.

This solid was dissolved in dioxane/H_2O (5 mL, 2:1), Na_2CO_3 (78 mg, 0.734 mmol, 1.5 equiv.) and benzyl chloroformate (70 μL, 0.489 mmol, 1.0 equiv.) were added and it was stirred for 20 h at room temperature. H_2O (50 mL) was added and it was extracted with CH_2Cl_2 (2 × 50 mL), dried over $MgSO_4$, and the solvent was removed under reduced pressure. Column chromatography on SiO_2 (cyclohexane/EtOAc 4:1) afforded the title compound (180 mg, 0.311 mmol, 64 % over 2 steps) as colorless oil.

R_f: 0.57 (Cyclohexane/EtOAc 2:1, $KMnO_4$)

^{1}H-NMR (400 MHz, DMSO-d$_6$, 90 °C): δ = 1.38 (m$_c$, 9 H, 3 × CH_3), 1.39 (m$_c$, 9 H, 3 × CH_3), 1.44−1.77 (m, 8 H, 4 × CH_2), 1.94−2.03 (m, 2 H, CH_2), 2.22−2.35 (m, 2 H, CH_2), 3.59 (m$_c$, 3 H, CO_2Me), 3.62−3.70 (m, 2 H, 2 × CH), 4.17−4.23 (m, 1 H, CH), 4.36 (d, *J* = 6.8 Hz, 1 H, NH), 4.59 (dd, *J* = 5.2, 6.8 Hz, 1 H, CH), 5.01 (d, *J* = 12.8 Hz, 1 H, CH_A), 5.19 (d, *J* = 12.8 Hz, 1 H, CH_B), 5.63 (s$_{br}$, 1 H, OH), 7.26−7.39 (m, 5 H, 5 × Ar-H).

^{13}C-NMR (100 MHz, DMSO-d$_6$, 90 °C): δ = 16.20, 20.72, 22.39, 24.91, 25.90, 27.18, 27.79, 32.07, 33.01, 50.39, 51.92, 54.08, 66.06, 72.67, 77.13, 80.50, 126.8, 127.1, 127.8, 136.5, 155.1, 155.2, 171.3, 172.7.

MS (ESI): *m/z* (%) = 423.2 (78) [M-Boc-*t*Bu+H]$^+$, 479.2 (100) [M-Boc+H]$^+$, 579.0 (56) [M+H]$^+$, 601.1 (42) [M+Na]$^+$.

HRMS (ESI) for $C_{30}H_{46}N_2O_9$: calcd. 579.3282 $[M+H]^+$, found 579.3394.

Benzyl (1*S*,4*R*,5*S*,6*R*)-4-(4-methoxy-4-oxobutyl)-2-oxo-5-[(triethylsilyl)oxy]-3,10-diazabicyclo[4.3.1]decane-10-carboxylate (15)

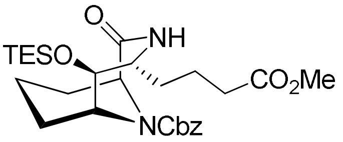

To a solution of **14** (1.50 g, 2.59 mmol) and 2,6-lutidine (12.1 mL, 104 mmol, 40 equiv.) in CH_2Cl_2 (52 mL) at 0 °C was added TESOTf (11.7 mL, 51.8 mmol, 20 equiv.) dropwise. After 1 h the cooling bath was removed and the reaction was stirred for 16 h at room temperature. Sat. aq. NH_4Cl solution (100 mL) was added and stirring was continued for 1 h. The organic phase was separated, the aqueous phase was extracted with CH_2Cl_2 (2 × 100 mL), the combined organic phases were dried over $MgSO_4$, and the solvent was evaporated under reduced pressure.
The obtained amino acid was dissolved in CH_2Cl_2 (25 mL) and the solution was added dropwise at room temperature over 30 min to a solution of HATU (985 mg, 2.59 mmol, 1.0 equiv.) and $(i\text{Pr})_2NEt$ (679 µL, 3.89 mmol, 1.5 equiv.) in CH_2Cl_2 (260 mL). Stirring was continued for further 20 h, following evaporation of the solvent under reduced pressure. The residue was taken up in CH_2Cl_2 (100 mL), washed with $CuSO_4$ solution (10 wt. %, 3 × 100 mL), dried over $MgSO_4$, and the solvent was evaporated under reduced pressure. Column chromatography on SiO_2 (cyclohexane/EtOAc 1:1) afforded the title compound (1.00 g, 1.93 mmol, 74% over 2 steps) as a slightly yellow oil.

The compound consists of two carbamate rotamers in a 1:1 ratio.

R_f: 0.49 (Cyclohexane/EtOAc 1:1, $KMnO_4$)

^{1}H-NMR (600 MHz, $CDCl_3$): δ = 0.58 (m_c, 6 H, 3 × CH_2), 0.94 (m_c, 9 H, 3 × CH_3), 1.24−1.89 (m, 8 H, 4 × CH_2), 2.07−2.27 (m, 4 H, 2 × CH_2), 2.95−3.08 (m, 1 H, CH), 3.63 (s, 1.5 H, CO_2Me), 3.65 (s, 1.5 H, CO_2Me), 4.09−4.14 (m, 1 H, CH), 4.39−4.43 (m, 0.5 H, CH), 4.46−4.50 (m, 0.5 H, CH), 4.97−5.01 (m, 0.5 H, CH), 5.06−5.09 (m, 0.5 H, CH), 5.10 (t, *J* = 12.0 Hz, 1 H, CH_2), 5.21 (d, *J* = 12.0 Hz, 0.5 H, CH_2), 5.24 (d, *J* = 12.0 Hz, 0.5 H, CH_2), 6.32−6.48 (m, 1 H, NH), 7.28−7.40 (m, 5 H, 5 × Ar-H).

^{13}C-NMR (125 MHz, $CDCl_3$): δ = 5.05, 6.90, 16.74, 23.37 (0.5 C), 23.63 (0.5 C), 26.32 (0.5 C), 27.01 (0.5 C),, 27.46 (0.5 C), 27.68 (0.5 C), 29.20, 33.82 (0.5 C), 33.89 (0.5 C), 51.68, 54.85 (0.5 C), 55.00 (0.5 C), 55.31 (0.5 C), 55.79, (0.5 C), 59.13, 67.86, 74.67,128.1 (0.5 C), 128.2 (0.5 C), 128.3 (0.5 C), 128.4 (0.5 C), 128.7, 136.5, 154.9 (0.5 C), 155.2 (0.5 C), 173.8 (0.5 C), 173.9 (0.5 C), 175.3 (0.5 C), 175.4 (0.5 C).

MS (ESI): *m/z* (%) = 519.2 (100) $[M+H]^+$, 1036.8 (18) $[2M+H]^+$, 1060.0 (6) $[2M+Na]^+$.

HRMS (ESI) for $C_{27}H_{42}N_2O_6Si$: calcd. 405.2026 $[M-TES+H]^+$, found 405.2098.

Benzyl (1*S*,4*R*,5*S*,6*R*)-5-methoxy-4-(4-methoxy-4-oxobutyl)-3-methyl-2-oxo-3,10-diazabicyclo[4.3.1]decane-10-carboxylate (16)

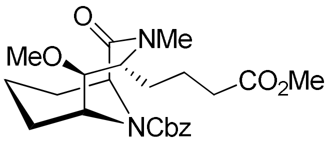

To a solution of **15** (300 mg, 0.578 mmol) in THF (3 mL) was added TBAF (1 M in THF, 636 µL, 0.636 mmol, 1.1 equiv.) and the solution was stirred for 1 h at room temperature. Removal of the solvent under reduced pressure and column chromatography on SiO_2 (EtOAc) afforded the alcohol (192 mg, 0.475 mmol, 82 %) as a colorless solid.

To a solution of the alcohol (219 mg, 0.541 mmol) in DMF (2 mL) was added Ag_2O (627 mg, 2.71 mmol, 5.0 equiv.) and MeI (337 µL, 5.41 mmol, 10 equiv.) and the suspension was stirred for 20 h at room temperature. The mixture was filtered over Celite, washed with EtOAc (50 mL), the filtrate was washed with sat. aq. NaCl solution (2 × 50 mL), the organic phase was dried over $MgSO_4$, and the solvent removed under reduced pressure. Column chromatography on SiO_2 (cyclohexane/EtOAc 1:2) afforded the title compound (210 mg, 0.486 mol, 90 %) as colorless oil.

The compound consists of two carbamate rotamers in a 1:1 ratio.

R_f: 0.35 (Cyclohexane/EtOAc 1:3, $KMnO_4$)

^{1}H-NMR (800 MHz, $CDCl_3$): δ = 1.39−1.52 (m, 1 H, 0.5 × CH_2), 1.58−1.87 (m, 8 H, 4 × CH_2), 2.05−2.33 (m, 3 H, 1.5 × CH_2), 3.09 (s, 1.5 H, NMe), 3.11 (s, 1.5 H, NMe), 3.31−3.35 (m, 1 H, CH), 3.38 (s, 1.5 H, OMe), 3.38 (s, 1.5 H, OMe), 3.58−3.62 (m, 1 H, CH), 3.65 (s, 1.5 H, CO_2Me), 3.66 (s, 1.5 H, CO_2Me), 4.32−4.35 (m, 0.5 H, CH), 4.43−4.46 (m, 0.5 H, CH), 4.99−5.01 (m, 0.5 H, CH), 5.07−5.09 (m, 0.5 H, CH), 5.10 (d, *J* = 12.0 Hz, 1 H, CH_2), 5.17 (d, *J* = 12.0 Hz, 0.5 H, CH_2), 5.21 (d, *J* = 12.0 Hz, 0.5 H, CH_2), 7.29−7.38 (m, 5 H, 5 × Ar-H).

^{13}C-NMR (200 MHz, $CDCl_3$): δ = 16.85 (0.5 C), 16.86 (0.5 C), 24.71 (0.5 C), 24.77 (0.5 C), 27.08 (0.5 C), 27.31 (0.5 C), 27.71 (0.5 C), 28.37 (0.5 C), 30.28 (0.5 C), 30.31 (0.5 C), 34.06, 40.32 (0.5 C), 40.42 (0.5 C), 51.68 (0.5 C), 51.71 (0.5 C), 52.25 (0.5 C), 52.85 (0.5 C), 55.68 (0.5 C), 56.00 (0.5 C), 58.39 (0.5 C), 58.43 (0.5 C), 62.21 (0.5 C), 62.50 (0.5 C), 67.87 (0.5 C), 67.92 (0.5 C), 83.89 (0.5 C), 84.45 (0.5 C), 128.3 (0.5 C), 128.4 (0.5 C), 128.4 (0.5 C), 128.4 (0.5 C), 128.7 (0.5 C), 128.7 (0.5 C), 136.5, 154.8 (0.5 C), 155.0 (0.5 C), 172.5 (0.5 C), 172.8 (0.5 C), 173.7 (0.5 C), 173.8 (0.5 C).

MS (ESI): *m/z* (%) = 433.2 (100) $[M+H]^+$, 865.0 (50) $[2M+H]^+$, 886.7 (6) $[2M+Na]^+$.

HRMS (ESI) for $C_{23}H_{32}N_2O_6$: calcd. 433.2339 $[M+H]^+$, found 433.2422.

(1*R*,4*S*,8*R*,14*S*)-14-Methoxy-2-methyl-2,9-diazatricyclo[6.5.1.0^{4,9}]tetradecane-3,10-dione (1)

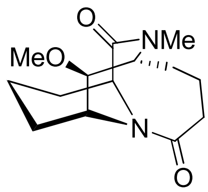

To a solution of **16** (233 mg, 0.539 mmol) in THF/H_2O (1 mL, 1:1) at 0 °C was added LiOH (39 mg, 1.62 mmol, 3.0 equiv.) and it was stirred for 4 h at 0 °C. H_2O (20 mL) was added and the pH was adjusted to 1 with 1 M aq. HCl solution. The aqueous solution was extracted with EtOAc (3 × 20 mL), the combined organic phases were dried over $MgSO_4$, and the solvent removed under reduced pressure to give the carboxylic acid (208 mg, 0.497 mmol, 92 %) as colorless semi-crystalline compound.

A suspension of the carboxylic acid (224 mg, 0.535 mmol) and Pd/C (22 mg, 10 wt. %) in EtOH (2 mL) was stirred for 2 h at room temperature under a H_2-atmosphere. The reaction mixture was filtered over

Celite, washed with EtOAc and the solvent was evaporated under reduced pressure to give the amino acid (134 mg, 0.471 mmol, 88 %) as slightly beige solid.

The amino acid (90 mg, 0.317 mmol) was suspended in CH_2Cl_2 (4mL) and the suspension was added dropwise at room temperature to a solution of HATU (119 mg, 0.317 mmol, 1.0 equiv.) and (iPr)$_2$NEt (83 μL, 0.478 mmol, 1.5 equiv.) in CH_2Cl_2 (32 mL). Stirring was continued for further 18 h, followed by evaporation of the solvent under reduced pressure. Column chromatography on SiO_2 (EtOAc) afforded the title compound (75 mg, 0.282 mmol, 89 %) as colorless crystals.

R_f: 0.17 (EtOAc, $KMnO_4$)

^{1}H-NMR (400 MHz, MeOH-d$_4$): δ = 1.60−1.88 (m, 7 H, 3.5 × CH_2), 2.21−2.54 (m, 4 H, 2 × CH_2), 2.99 (s, 3 H, NMe), 3.00−3.07 (m, 1 H, CH), 3.47 (s, 3 H, OMe), 3.84−3.93 (m, 2 H, 2 × CH), 4.10−4.16 (m, 1 H, CH), 5.21−5.26 (m, 1 H, CH).

^{13}C-NMR (100 MHz, MeOH-d$_4$): δ = 16.94, 19.75, 26.51, 27.43, 28.41, 33.86, 38.76, 54.58, 55.77, 58.95, 59.62, 84.48, 175.2, 177.5.

MS (ESI): *m/z* (%) = 267.1 (84) [M+H]$^+$, 533.0 (100) [2M+H]$^+$.

HRMS (ESI) for $C_{14}H_{22}N_2O_3$: calcd. 267.1709 [M+H]$^+$, found 267.1749.

^{1}H- and ^{13}C-Spectra of the Compounds
Compound 7

^{1}H-NMR, 400 MHz, DMSO-d$_6$

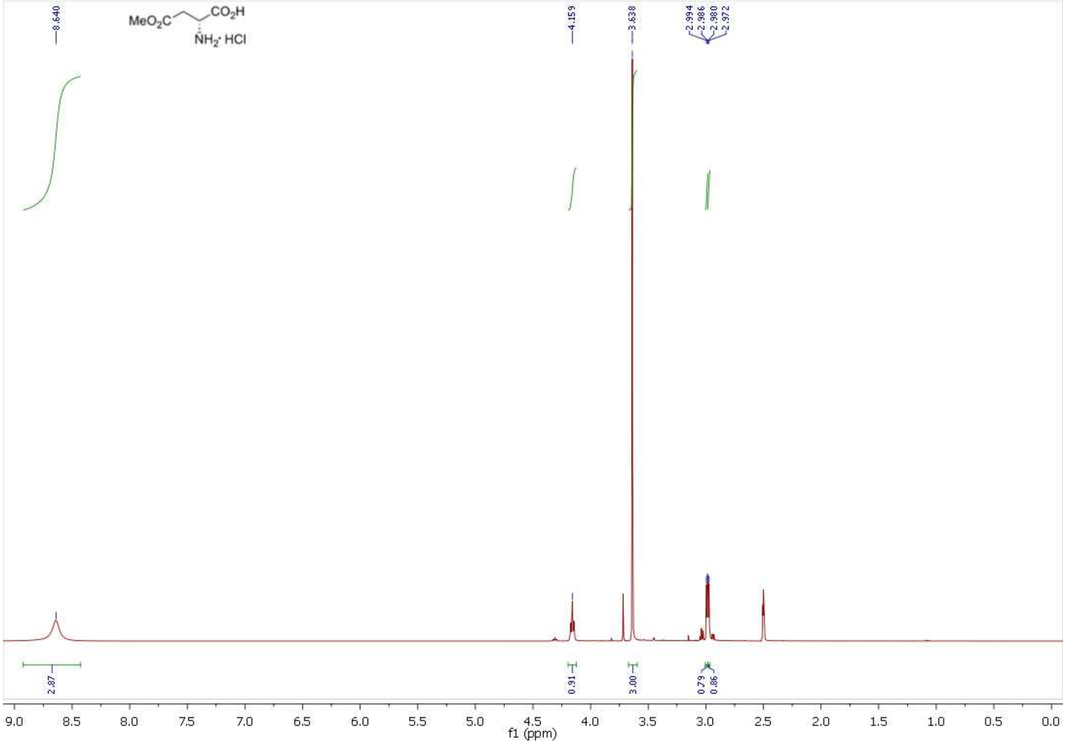

^{13}C-NMR, 100 MHz, DMSO-d$_6$

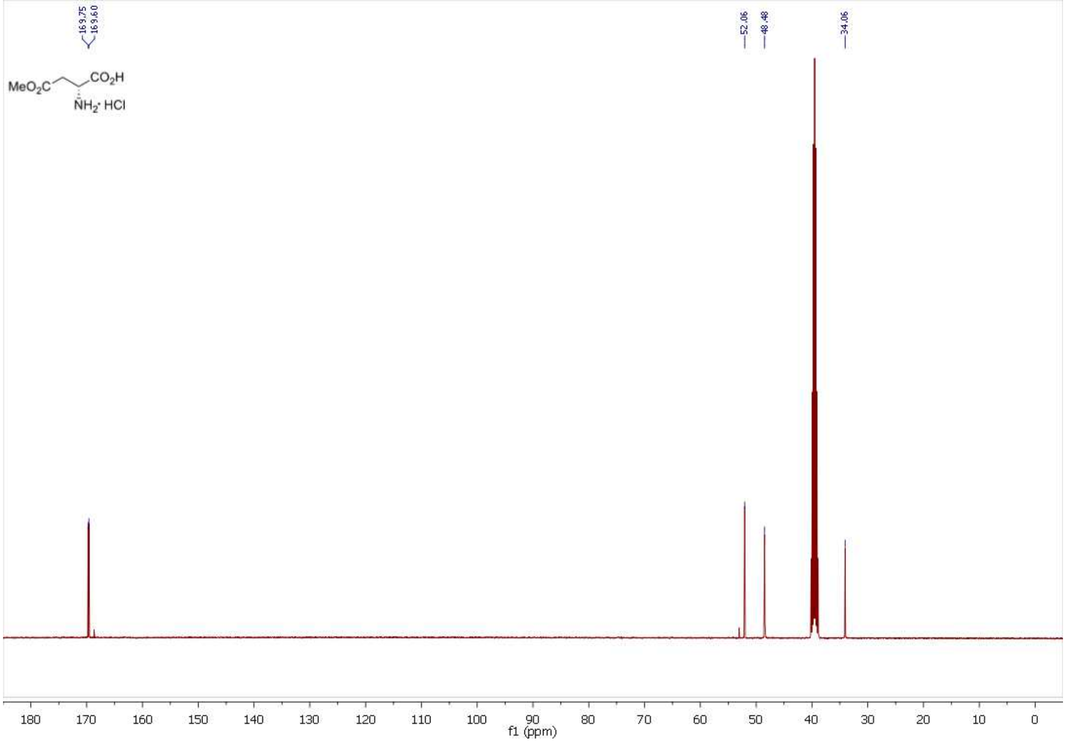

Compound 8

^{1}H-NMR, 300 MHz, $CDCl_3$

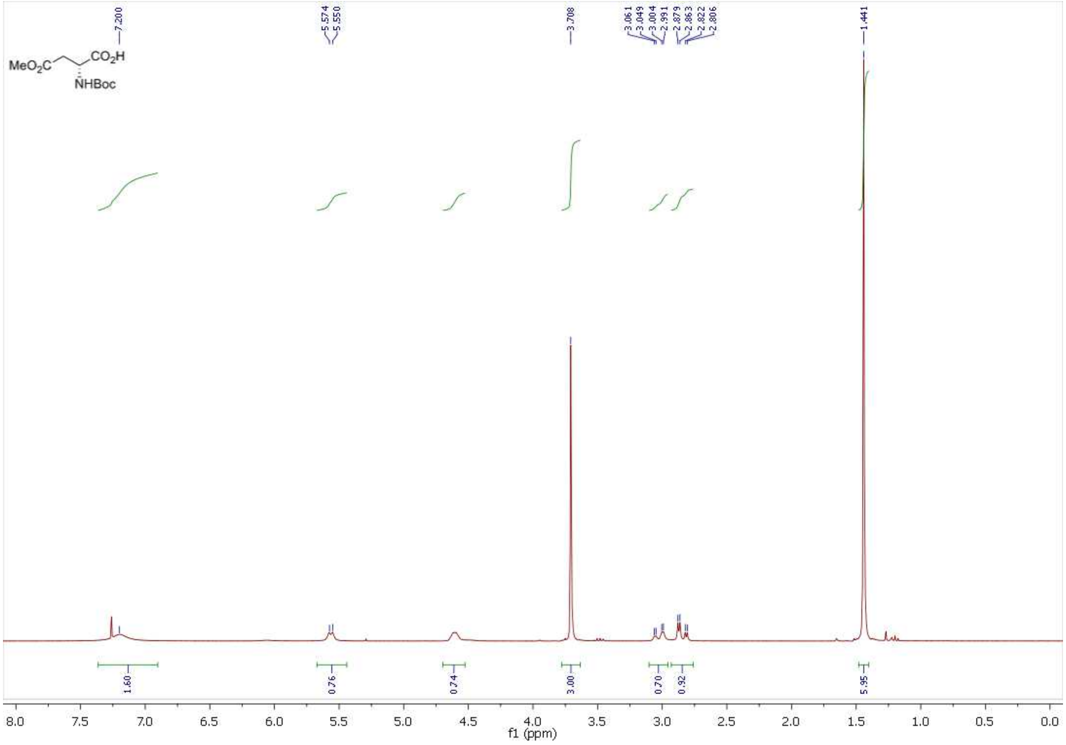

^{13}C-NMR, 75.5 MHz, $CDCl_3$

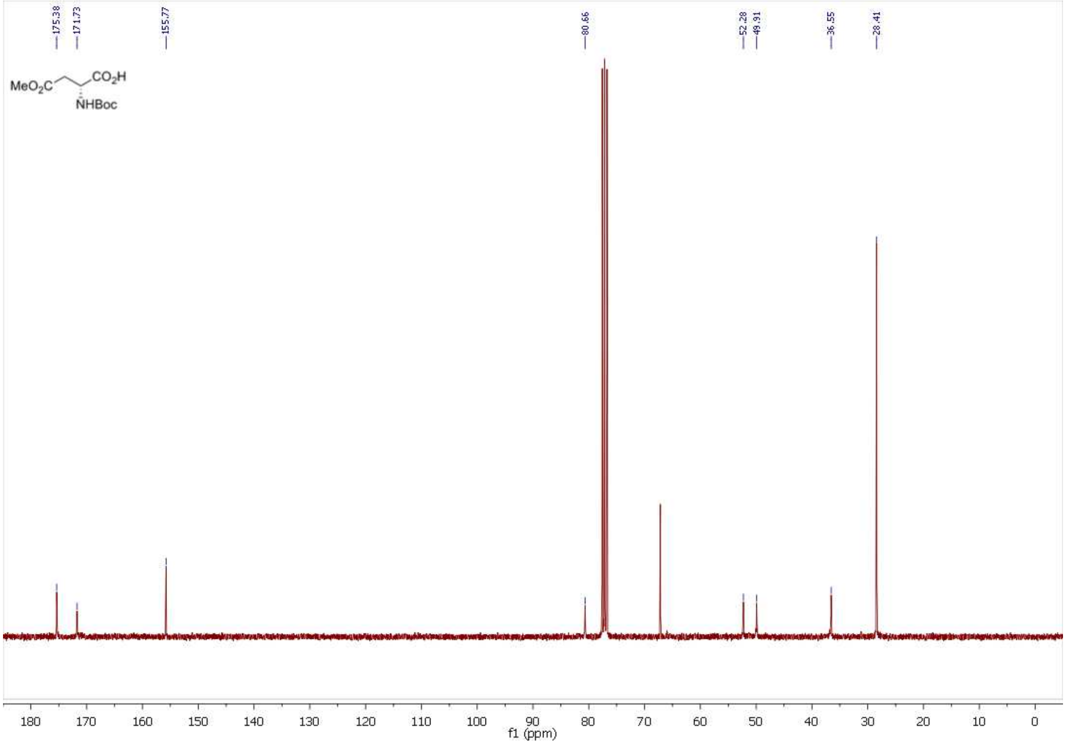

Compound 9

^{1}H-NMR, 400 MHz, DMSO-d$_6$

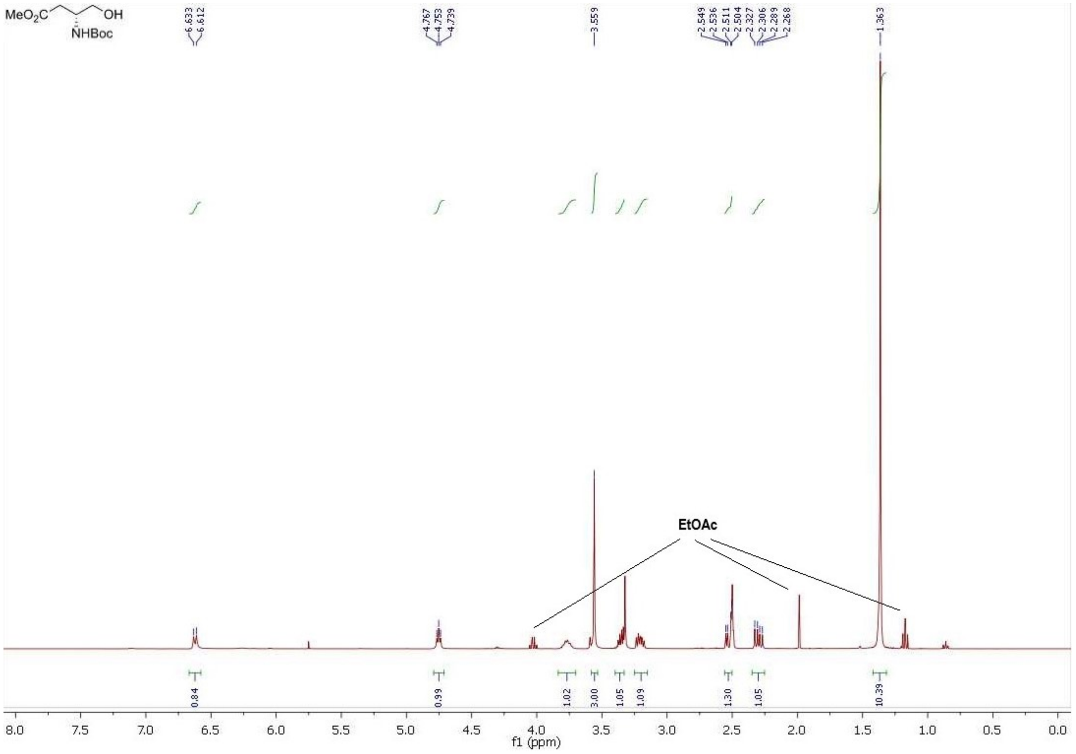

^{13}C-NMR, 100 MHz, DMSO-d$_6$

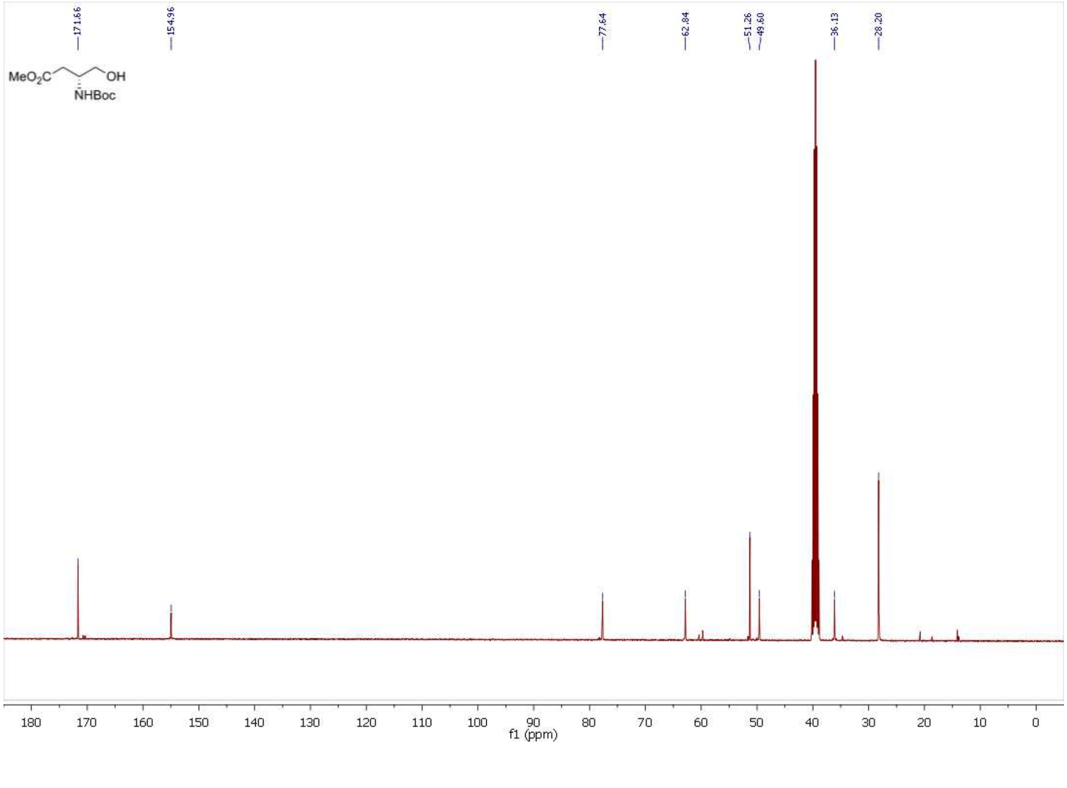

Compound 10

^{1}H-NMR, 400 MHz, $CDCl_3$

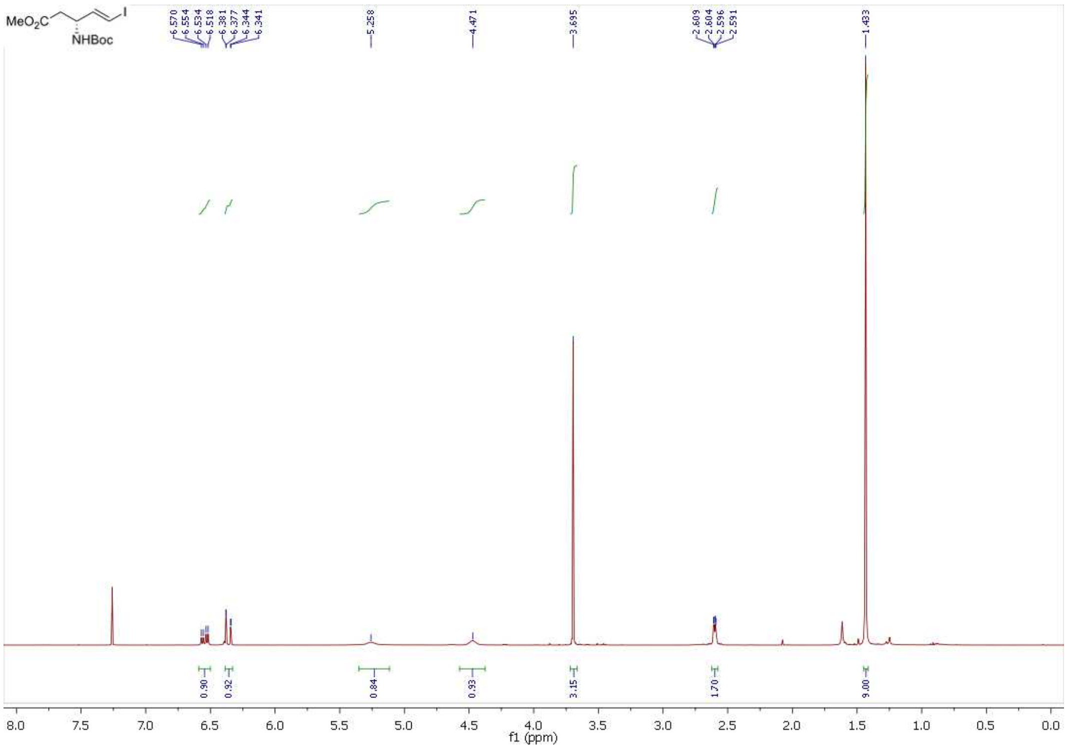

^{13}C-NMR, 100 MHz, $CDCl_3$

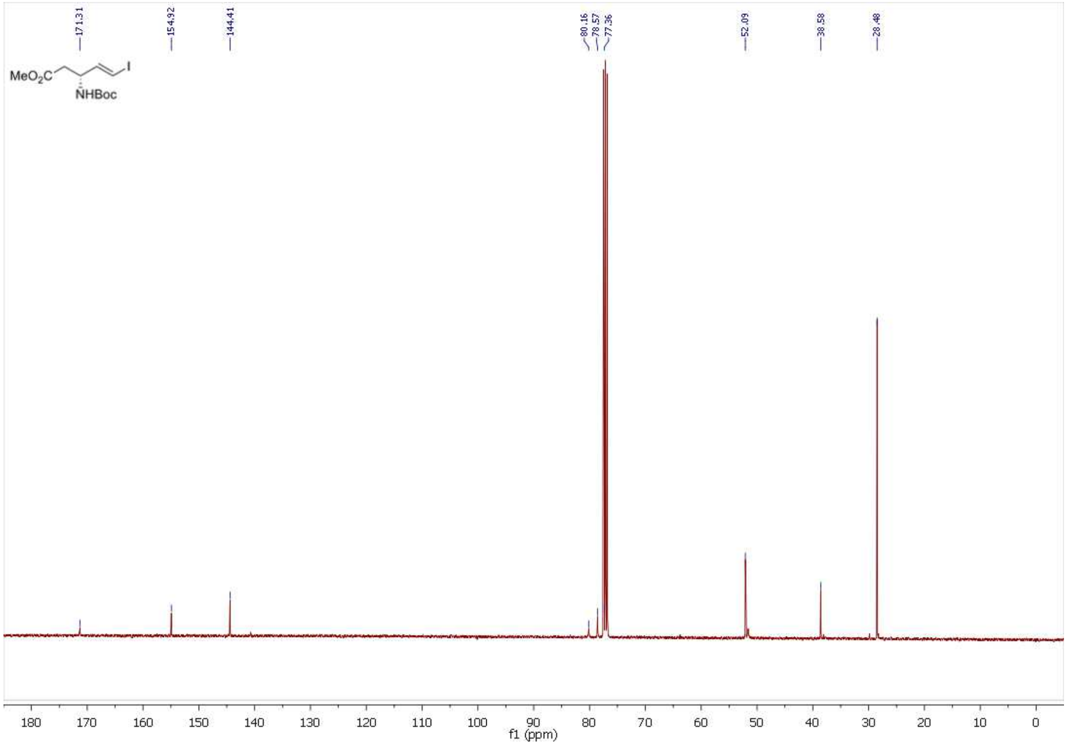

Compound 12

^{1}H-NMR, 300 MHz, $CDCl_3$

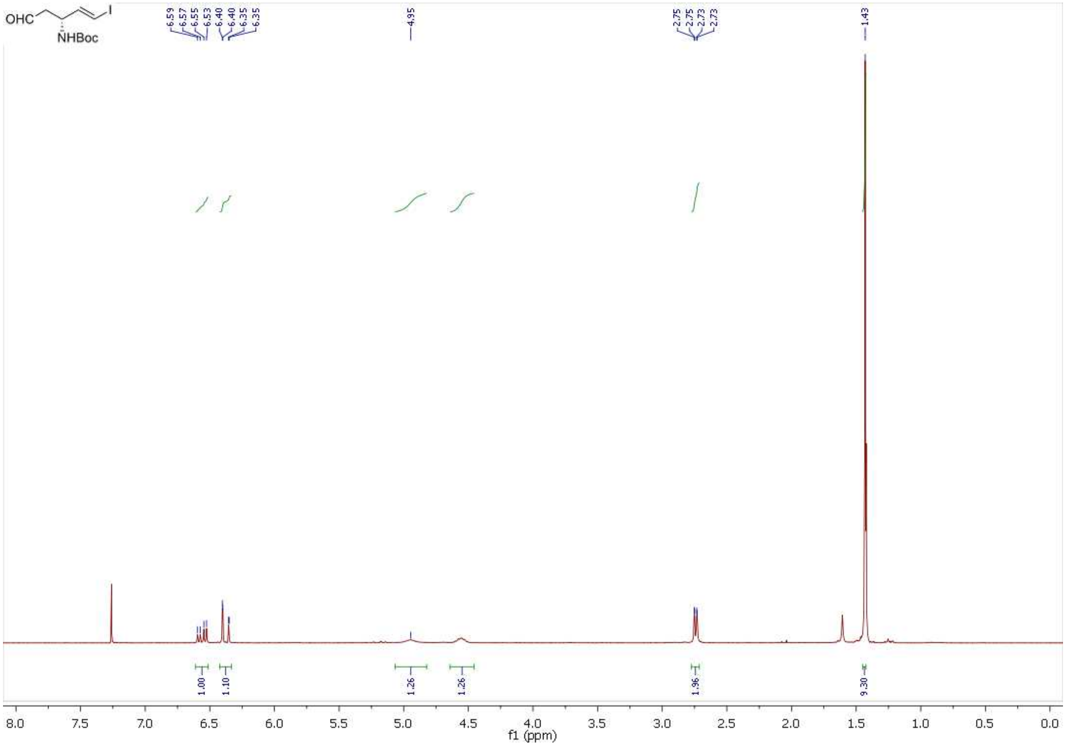

^{13}C-NMR, 75.5 MHz, $CDCl_3$

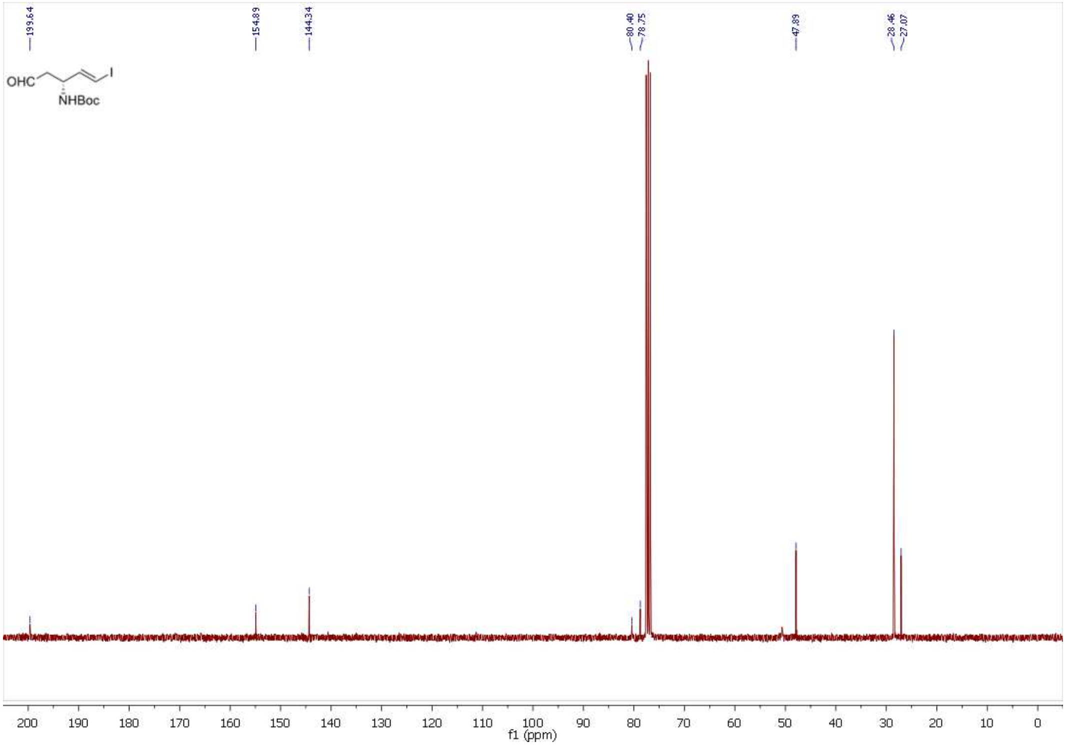

Compound 3

^{1}H-NMR, 600 MHz, $CDCl_3$

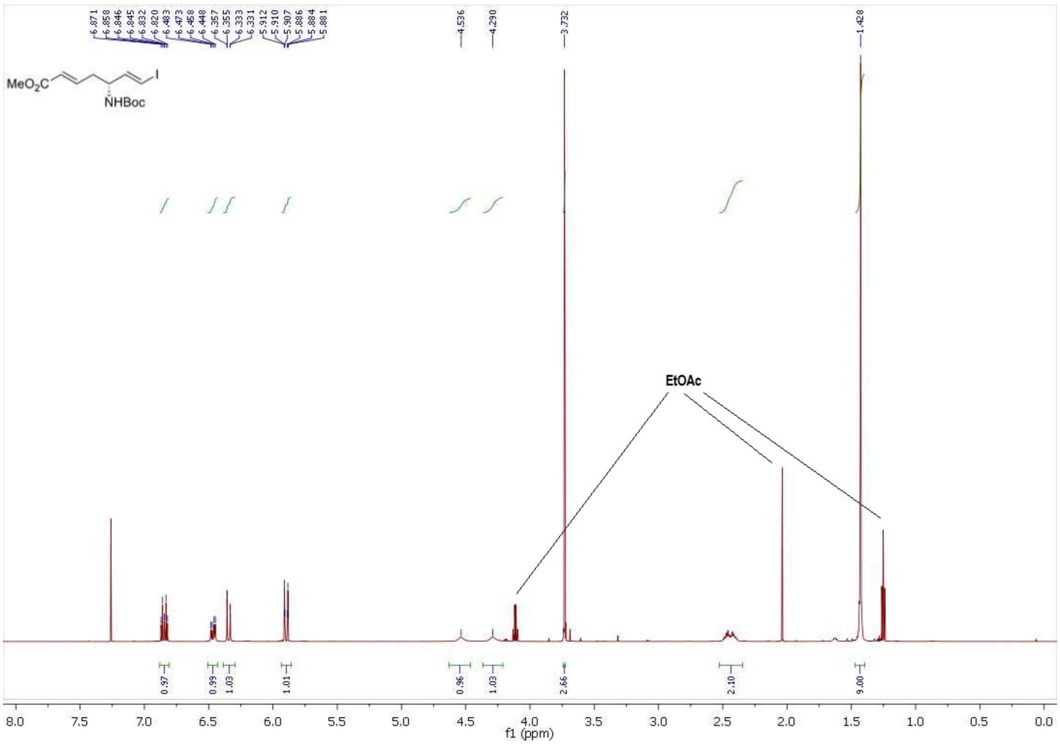

^{13}C-NMR, 125 MHz, $CDCl_3$

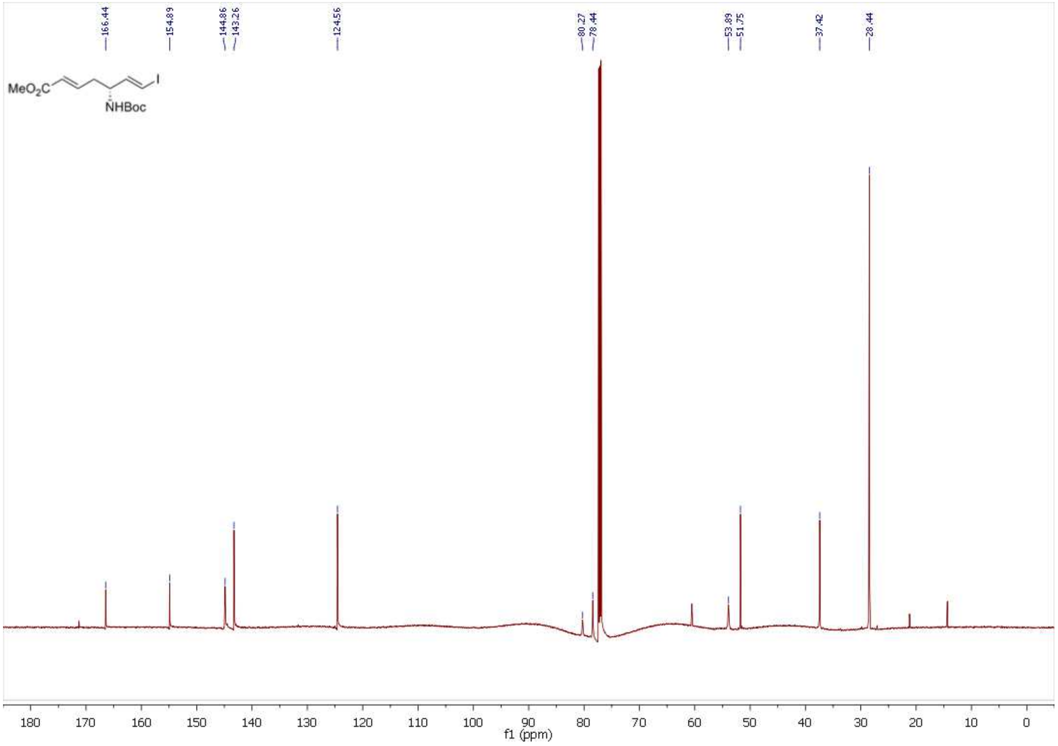

Compound 13

^{1}H-NMR, 600 MHz, $CDCl_3$

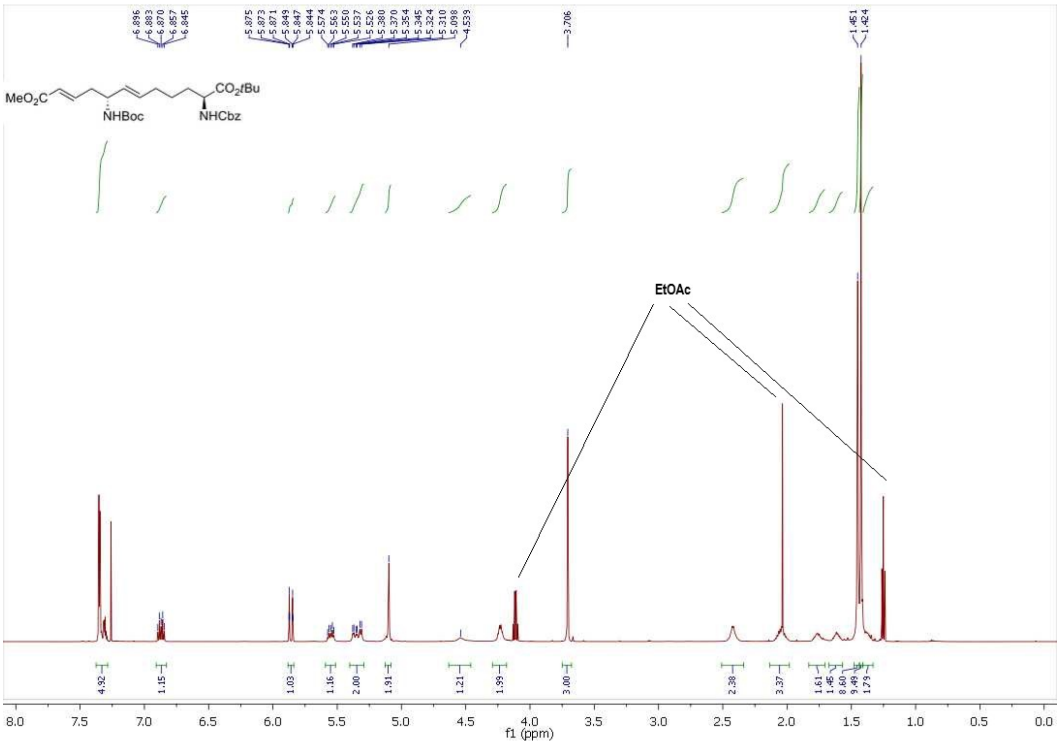

^{13}C-NMR, 125 MHz, $CDCl_3$

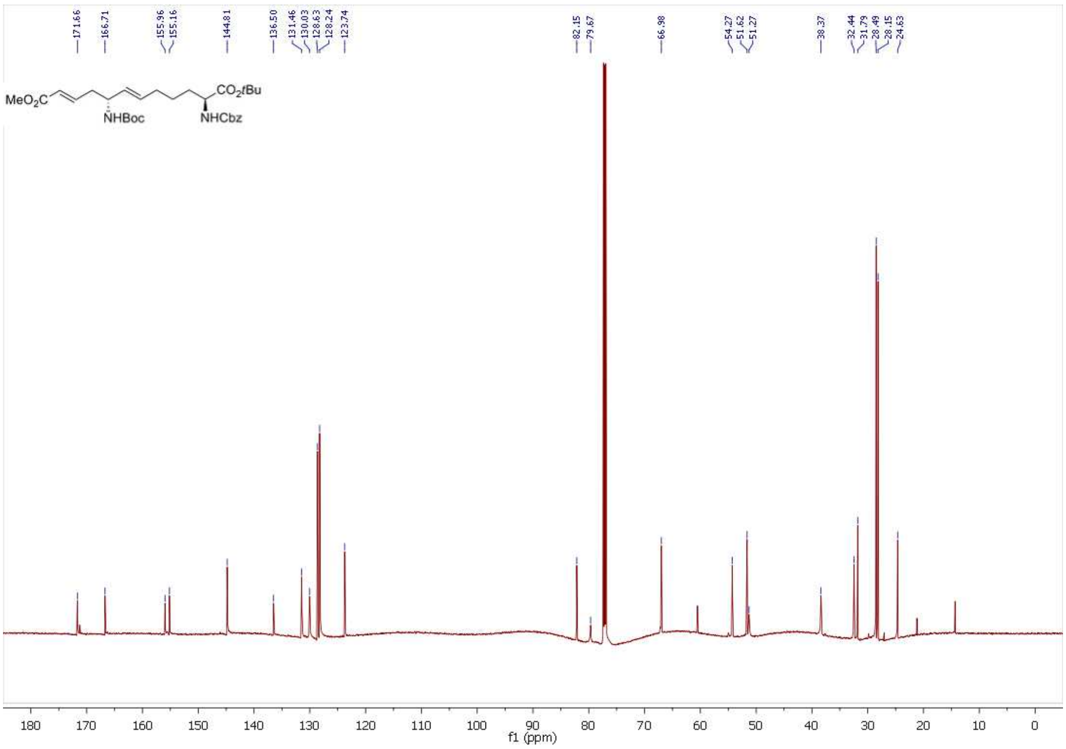

Compound 2

^{1}H-NMR, 600 MHz, $CDCl_3$

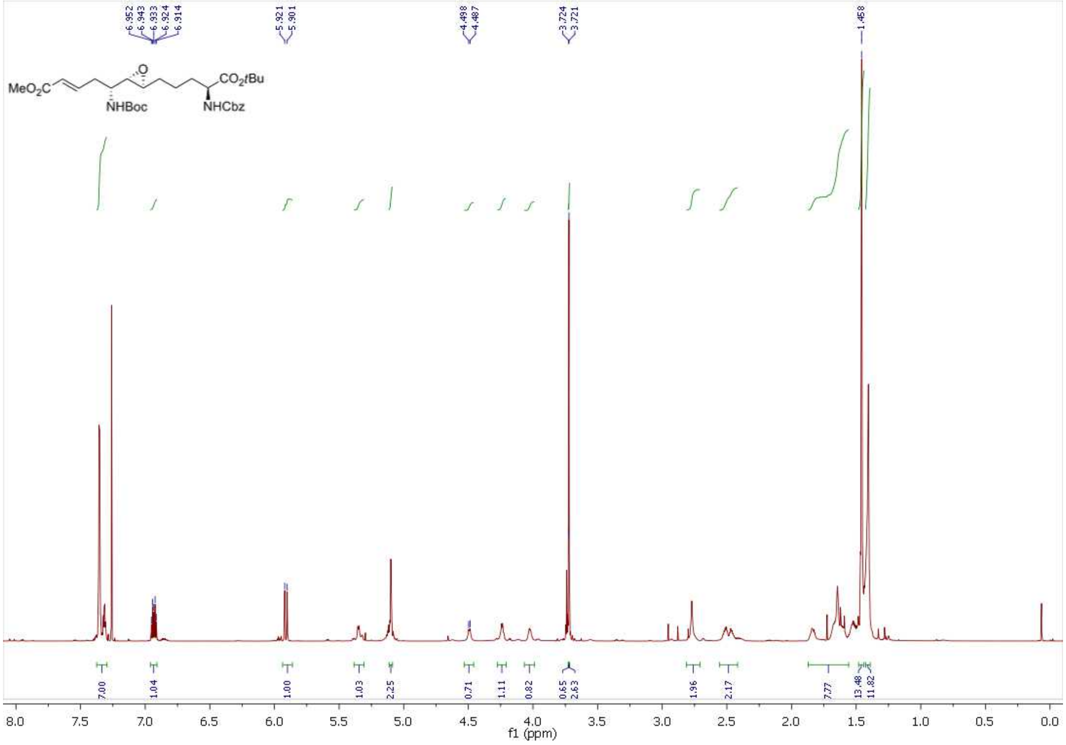

^{13}C-NMR, 125 MHz, $CDCl_3$

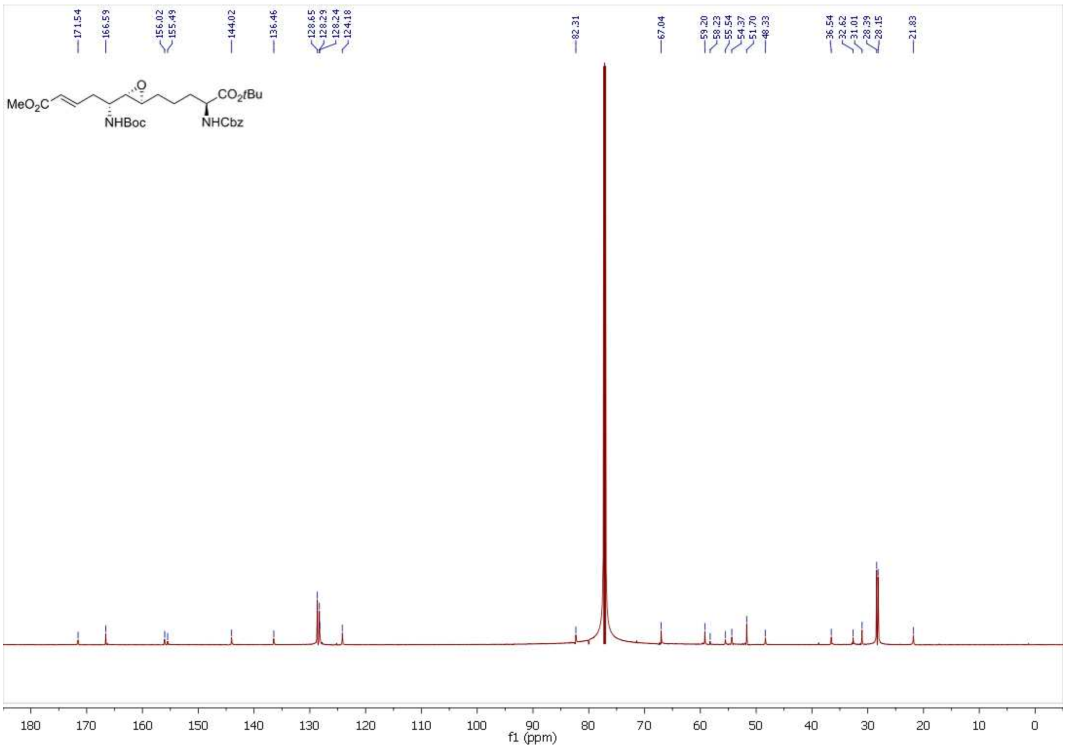

Compound 14

^{1}H-NMR, 400 MHz, DMSO-d$_6$, 90 °C

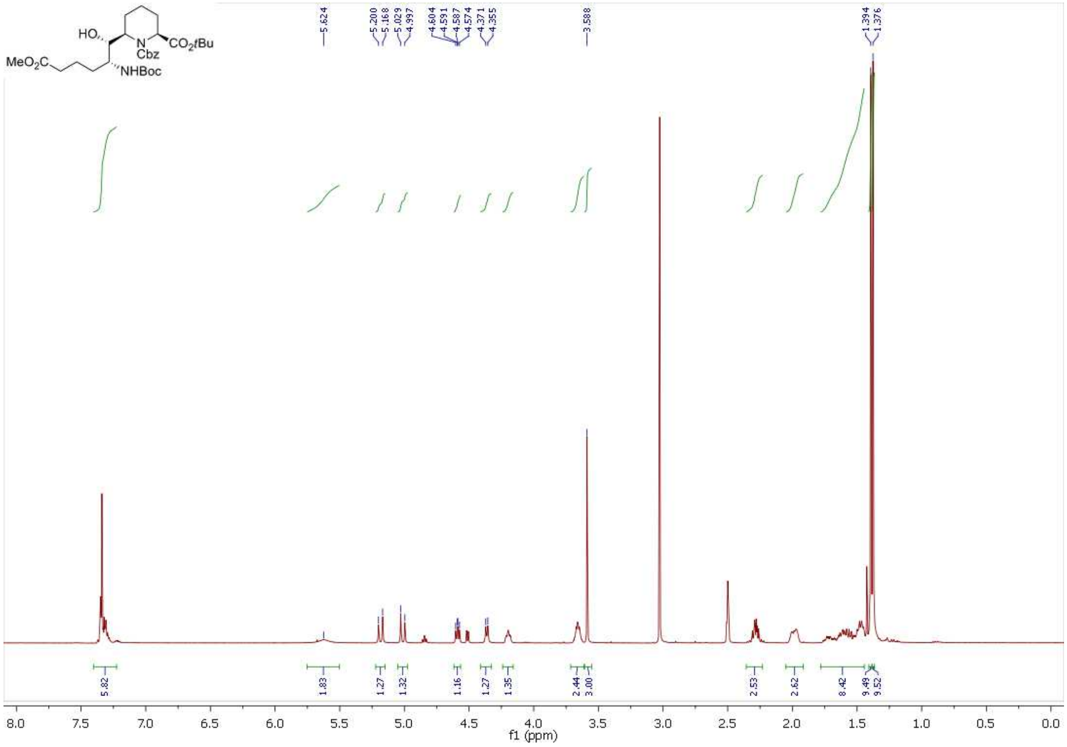

^{13}C-NMR, 100 MHz, DMSO-d$_6$, 90 °C

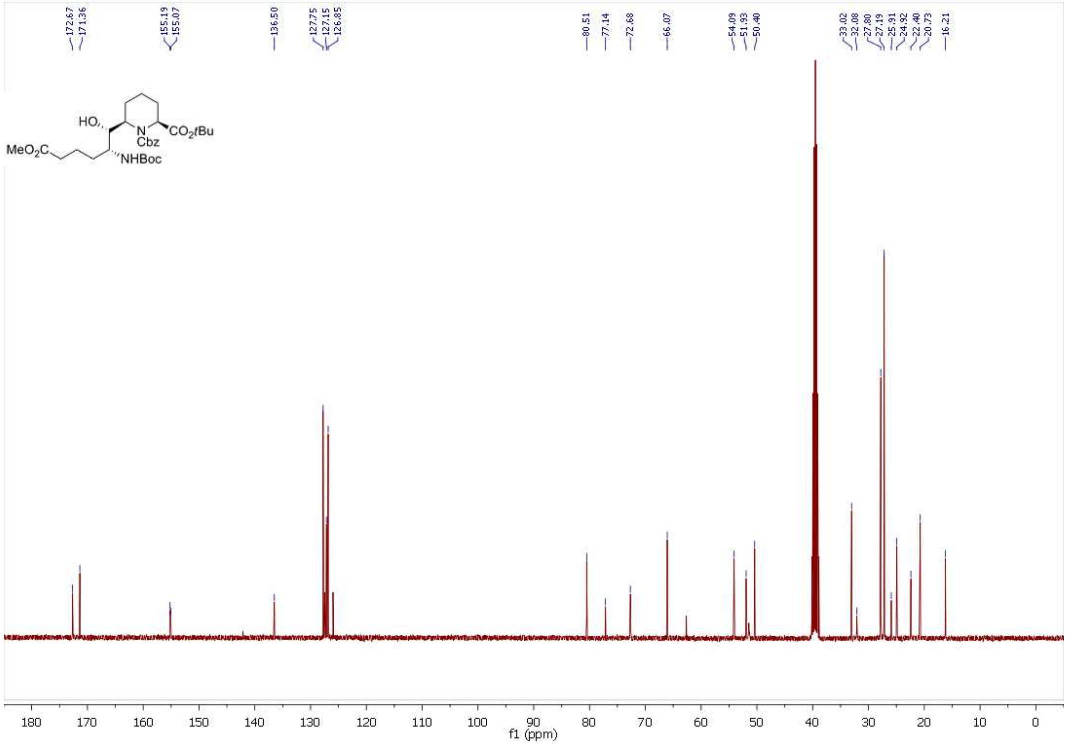

Compound 15

^{1}H-NMR, 600 MHz, $CDCl_3$

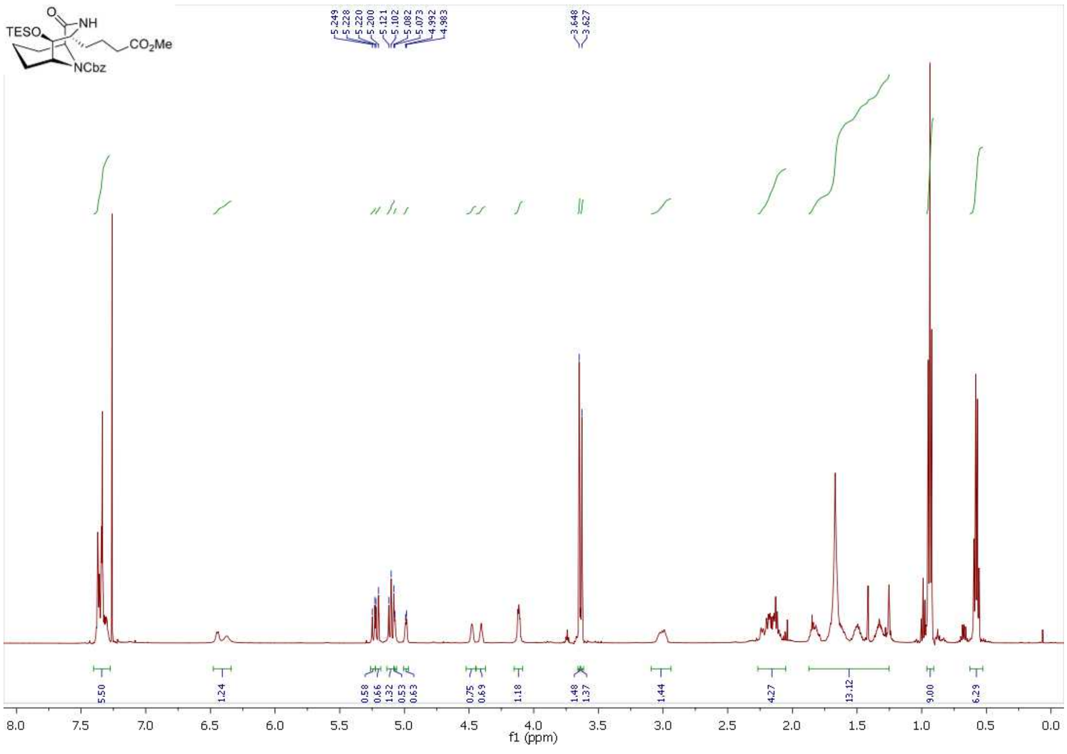

^{13}C-NMR, 125 MHz, $CDCl_3$

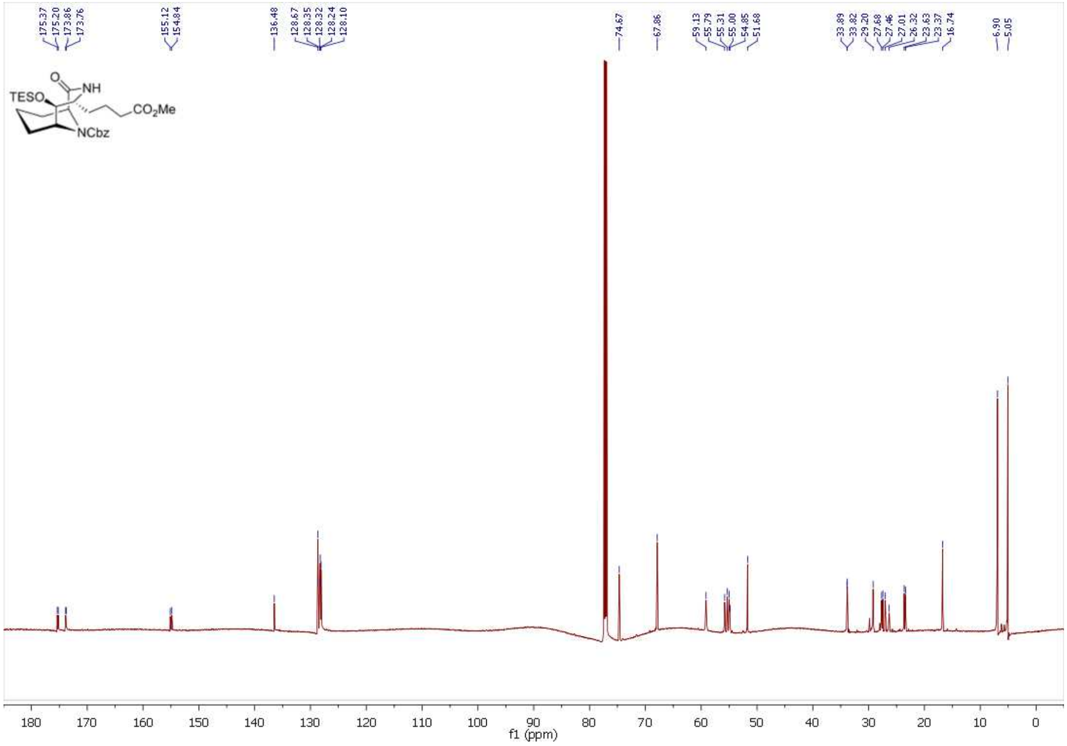

Compound 16

^{1}H-NMR, 800 MHz, $CDCl_3$

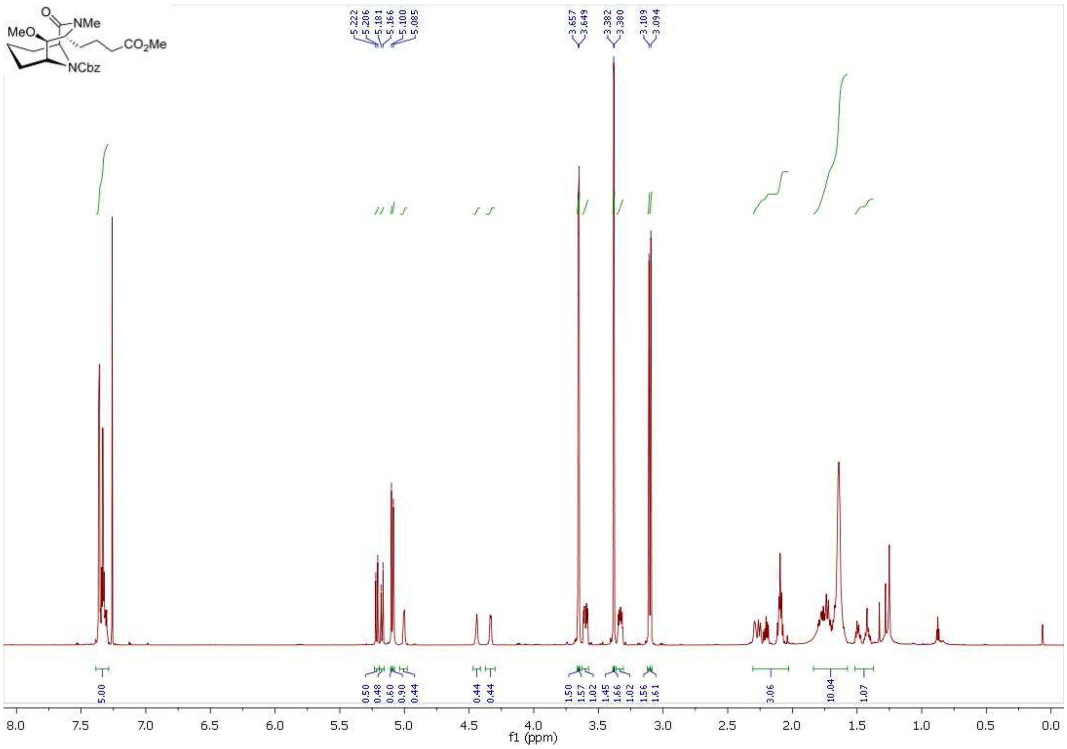

^{13}C-NMR, 200 MHz, $CDCl_3$

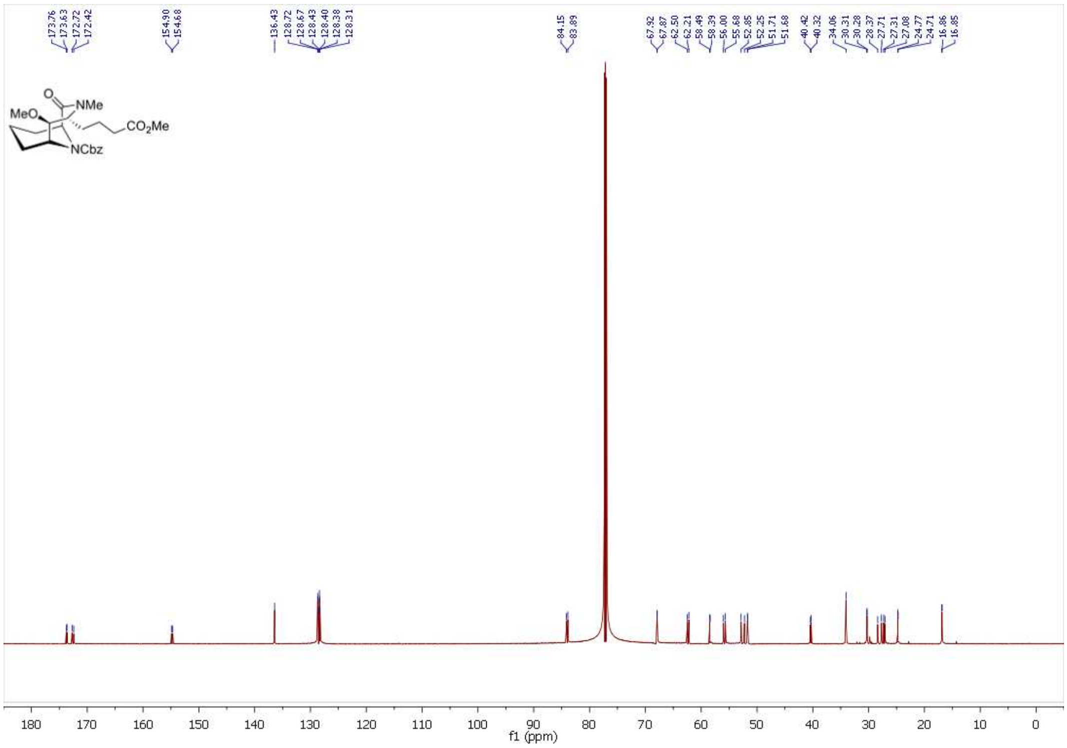

Compound 1

^{1}H-NMR, 400 MHz, MeOH-d$_4$

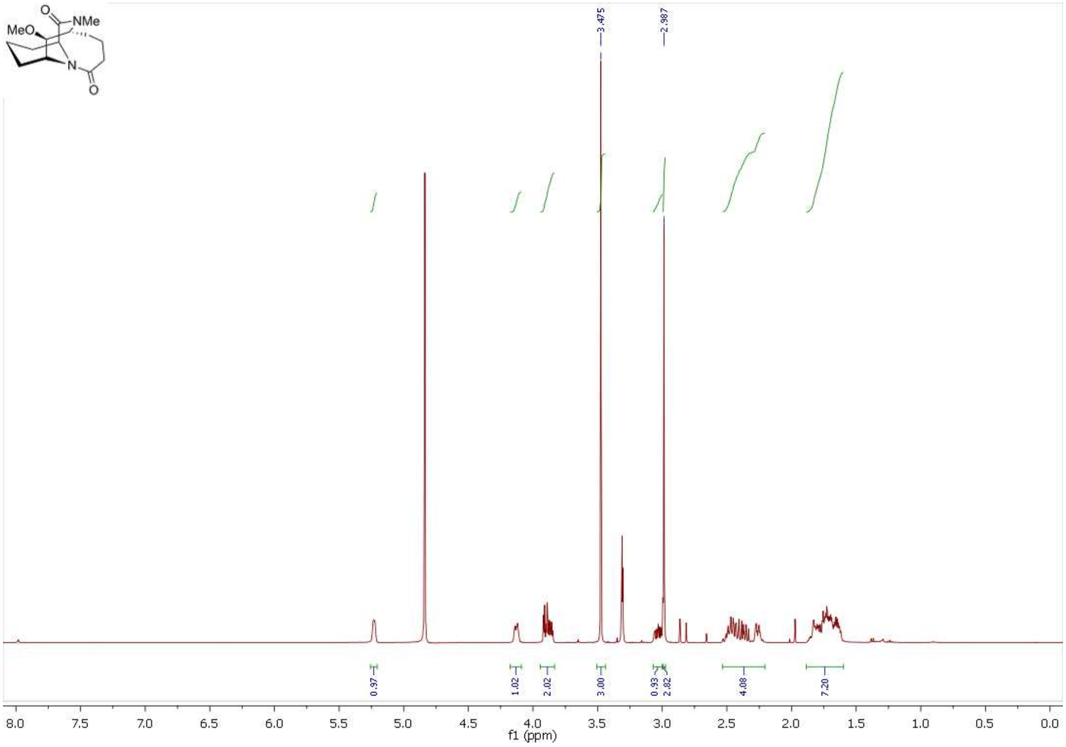

^{13}C-NMR, 100 MHz, MeOH-d$_4$

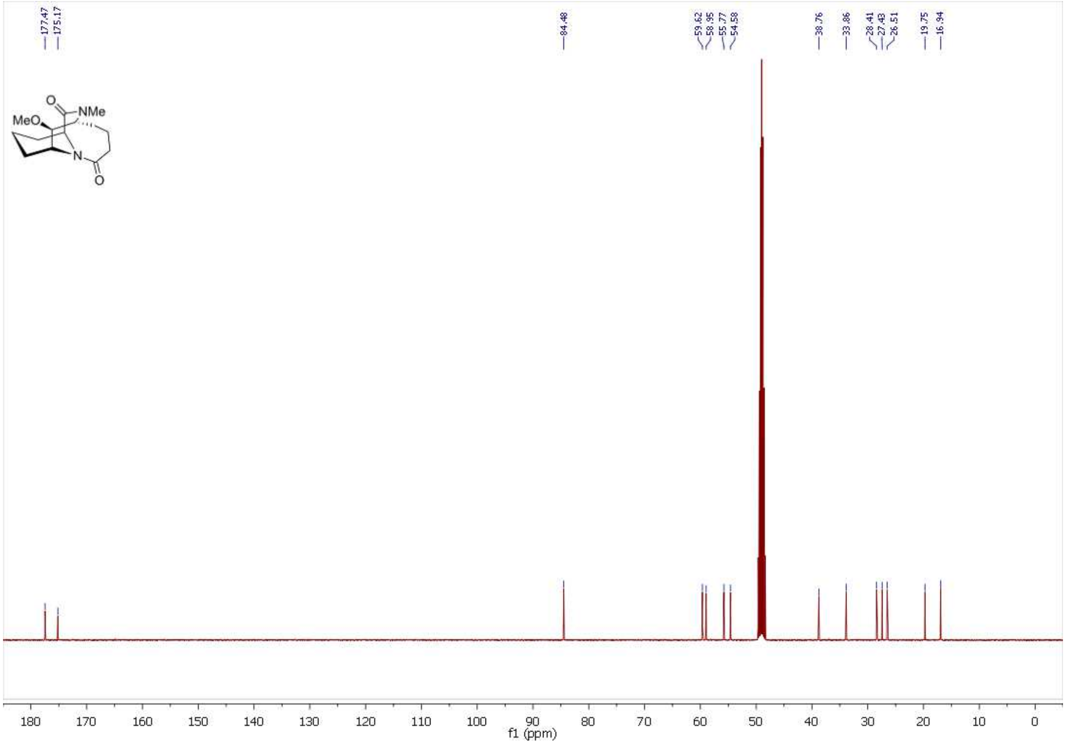

Crystallographic Data of 1

Crystallographic data: C14H22N2O3_H2O

	**1**
net formula	$C_{14}H_{24}N_2O_4$
M_r/g mol^{-1}	284.35
crystal size/mm	0.100 × 0.070 × 0.050
T/K	173(2)
radiation	MoKα
diffractometer	'Bruker D8Venture'
crystal system	monoclinic
space group	'P 21'
a/Å	8.2276(6)
b/Å	12.1866(9)
c/Å	8.2513(7)
α/°	90
β/°	116.603(2)
γ/°	90
V/Å^3	739.74(10)
Z	2
calc. density/g cm^{-3}	1.277
μ/mm^{-1}	0.093
absorption correction	multi-scan
transmission factor range	0.8931–0.9585
refls. measured	9065
R_{int}	0.0247
mean σ(I)/I	0.0273
θ range	3.228–26.39
observed refls.	2779
x, y (weighting scheme)	0.0331, 0.1292
hydrogen refinement	mixed
Flack parameter	0.2(3)
refls in refinement	3030

parameters	191
restraints	1
$R(F_{obs})$	0.0318
$R_w(F^2)$	0.0723
S	1.064
shift/error$_{max}$	0.001
max electron density/e Å^{-3}	0.201
min electron density/e Å^{-3}	−0.128

C-H: constr, O-H: refall.

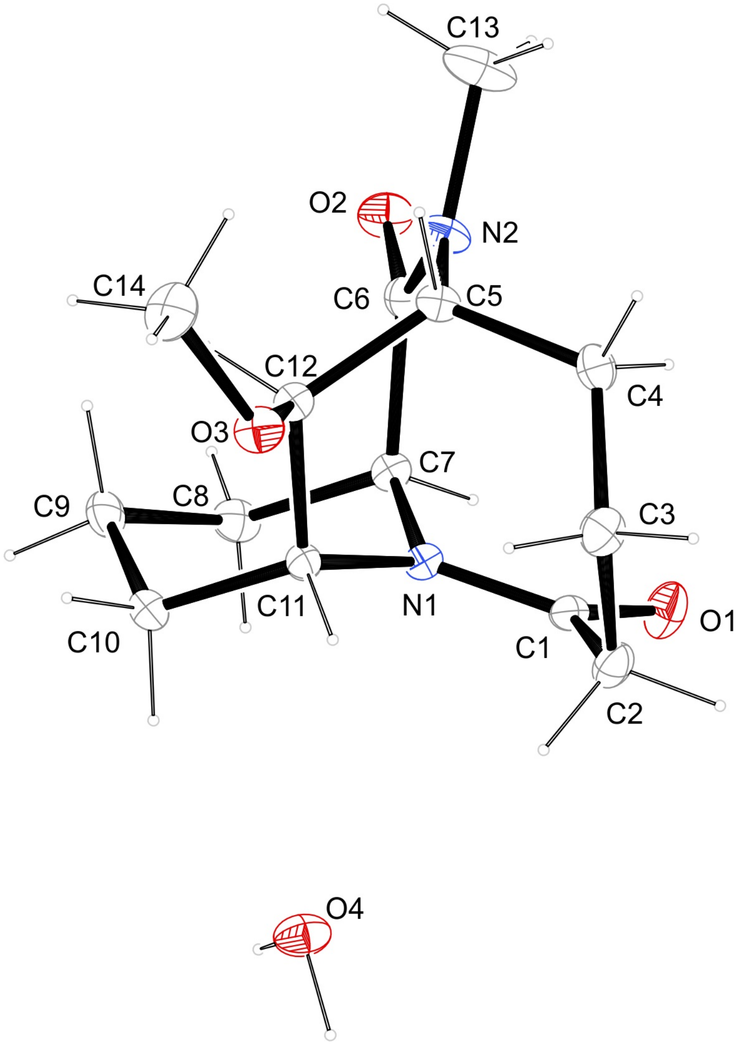

Scheme 1. Crystal structure of **1**.
